# Time‐resolved mRNA and miRNA expression profiling reveals crucial coregulation of molecular pathways involved in epithelial–pneumococcal interactions

**DOI:** 10.1111/imcb.12371

**Published:** 2020-07-20

**Authors:** Haiyan Li, Li Lin, Lei Chong, Shuge Gu, Shunhang Wen, Gang Yu, Xiaoguang Hu, Lin Dong, Hailin Zhang, Changchong Li

**Affiliations:** ^1^ Department of Pediatric Pulmonology The Second Affiliated Hospital and Yuying Children’s Hospital of Wenzhou Medical University Wenzhou China

**Keywords:** expression profiling, host–pathogen interactions, immune response, lung epithelial cells, network analysis, *Streptococcus pneumoniae*

## Abstract

*Streptococcus pneumoniae* is a major causative agent of pneumonia worldwide and its complex interaction with the lung epithelium has not been thoroughly characterized. In this study, we exploited both RNA‐sequencing and microRNA (miRNA)‐sequencing approaches to monitor the transcriptional changes in human lung alveolar epithelial cells infected by *S. pneumoniae* in a time‐resolved manner. A total of 1330 differentially expressed (DE) genes and 45 DE miRNAs were identified in all comparisons during the infection process. Clustering analysis showed that all DE genes were grouped into six clusters, several of which were primarily involved in inflammatory or immune responses. In addition, target gene enrichment analyses identified 11 transcription factors that were predicted to link at least one of four clusters, revealing transcriptional coregulation of multiple processes or pathways by common transcription factors. Notably, pharmacological treatment suggested that phosphorylation of p65 is important for optimal transcriptional regulation of target genes in epithelial cells exposed to pathogens. Furthermore, network‐based clustering analysis separated the DE genes negatively regulated by DE miRNAs into two functional modules (M1 and M2), with an enrichment in immune responses and apoptotic signaling pathways for M1. Integrated network analyses of potential regulatory interactions in M1 revealed that multiple DE genes related to immunity and apoptosis were regulated by multiple miRNAs, indicating the coordinated regulation of multiple genes by multiple miRNAs. In conclusion, time‐series expression profiling of messenger RNA and miRNA provides a wealth of information for global transcriptional changes, and offers comprehensive insight into the molecular mechanisms underlying host–pathogen interactions.

## Graphical abstract

In this study, we applied RNA‐sequencing technology to investigate the time‐resolved gene expression profiles of microRNAs (miRNAs) and messenger RNAs (mRNAs) in human lung alveolar epithelial cells in response to *Streptococcus pneumoniae* up to 8 h postinfection. Through a range of bioinformatics and function‐related analyses, we found several functional clusters and key regulators associated with inflammatory and immune responses. Finally, we built a regulatory network among differentially expressed miRNAs and differentially expressed target mRNAs to investigate the potential biological function or relevance during infection of miRNA–mRNA interactions.

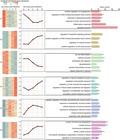

## Introduction

The epithelial surfaces of the lung, with direct contact to the atmospheric environment, provide an easy entry point for microbes and, therefore, are prone to infectious attack by a diverse range of microbial pathogens, including bacteria, fungi, viruses and pathogenic protozoa.^1^, ^2^ Among these microbial pathogens, the Gram‐positive bacterium *Streptococcus pneumoniae* is the most common causative pathogen of community‐acquired pneumonia, a major cause of infant mortality worldwide.^3^ *S. pneumoniae* primarily colonizes the host nasopharynx by adhering to mucosal surfaces of the upper airway.^4^, ^5^ An altered external environment or weakened host immunity allows *S. pneumoniae* in the upper respiratory tract to invade easily the lower airways, causing pneumonia and provoking host inflammatory and immune responses.^3^ Moreover, lung epithelial cells have been demonstrated to prevent entry and foster removal of pathogens by accumulating a physical mucus barrier, producing antimicrobial peptides and releasing a wide range of proinflammatory cytokines in response to *S. pneumoniae*.^1^, ^6^ Thus, dissecting the host immune responses to pneumococcal infection in detail will provide valuable information for the prevention and treatment of bacterial infections.^1^, ^2^

During infection, a number of common host transcriptional response genes have been reported to be involved in host cellular responses to microbial pathogens in the lungs.^7^, ^8^, ^9^ These mainly include genes that mediate inflammation (e.g. *CCL3*, *CCL4*, *CXCL1* and *CXCL2*), genes that activate immune responses (e.g. *BCL3*, *JUNB* and *TRAF1*) and genes that limit immune responses (e.g. *NFKBIA*, *TNFAIP3* and *BIRC3*).^9^ In addition, epithelial cell apoptosis could be efficiently activated to remove infected lung epithelial cells and pathogens as the infection progresses, indicating the role of cell apoptosis in the inhibition of inflammation.^2^ Increasing evidence has also demonstrated that transcription factors (TFs) are key regulators in modulating the activation of host immune responses and production of several anti‐inflammatory cytokines during pathogen infection.^7^, ^10^ Although studies have addressed the changes in expression of genes involved in cell apoptosis and transcription in the host during infection, a comprehensive profile for these processes in host lung epithelial cells during *S. pneumoniae* infection remains to be elucidated.

Recently, the application of transcriptome sequencing technology, including microarray and RNA‐sequencing (RNA‐seq), has greatly facilitated genome‐wide scanning of host transcriptional responses to *S. pneumoniae* using various *in vitro* and *in vivo* infection models.^4^, ^11^, ^12^, ^13^ Global transcriptional profiling of Detroit cells during infection of *S. pneumoniae* showed that over one‐third of induced genes were mainly involved in transcriptional regulation and signal transduction, in particular MAPK signaling pathways.^14^ Microarray and bioinformatics analyses of mouse whole lung have indicated the involvement of interleukin (IL)‐17A/IL‐17F signaling and lipid metabolism in acute *S. pneumoniae* infection.^12^ Reactive oxygen species produced by *S. pneumoniae* can activate the glutathione‐dependent reactive oxygen detoxification pathway in human lung epithelial cells, as suggested by transcriptome changes observed using time‐resolved dual RNA‐seq analysis.^11^ In addition, recent transcriptome studies have shown the critical role of microRNAs (miRNAs) in modulating cell apoptosis, immunity and external stimulation.^15^, ^16^ However, integrated analyses of miRNA–messenger RNA (mRNA) transcriptome changes have been scarcely reported in the time‐resolved infection of human lung epithelial cells by *S. pneumoniae*.

Serotype 3 encapsulated *S. pneumoniae* was an important cause of invasive pneumococcal disease with severe complications, including parapneumonic empyema and hemolytic uremic syndrome.^17^, ^18^ However, only few studies have investigated the host transcriptomic responses to this strain, and exploration of the response of the epithelium during infection by serotype 3 might provide new molecular insights into host–pathogen interactions. In this study, we applied RNA‐seq technology to investigate the time‐resolved gene expression profiles of miRNAs and mRNAs in human lung alveolar epithelial cells in response to *S. pneumoniae* up to 8 h postinfection (hpi). We performed quantitative reverse transcription‐PCR (qRT‐PCR) to further confirm the high‐quality data sets generated from RNA‐seq and miRNA‐seq. Through a range of bioinformatics and function‐related analyses, we found several functional clusters and key regulators associated with inflammatory and immune responses. Finally, we built a regulatory network among differentially expressed (DE) miRNAs and DE target mRNAs to investigate the potential biological function or relevance of miRNA–mRNA interactions during infection.

## Results

### RNA‐seq generates high‐quality data sets for differential expression analysis

**Figure 1:**
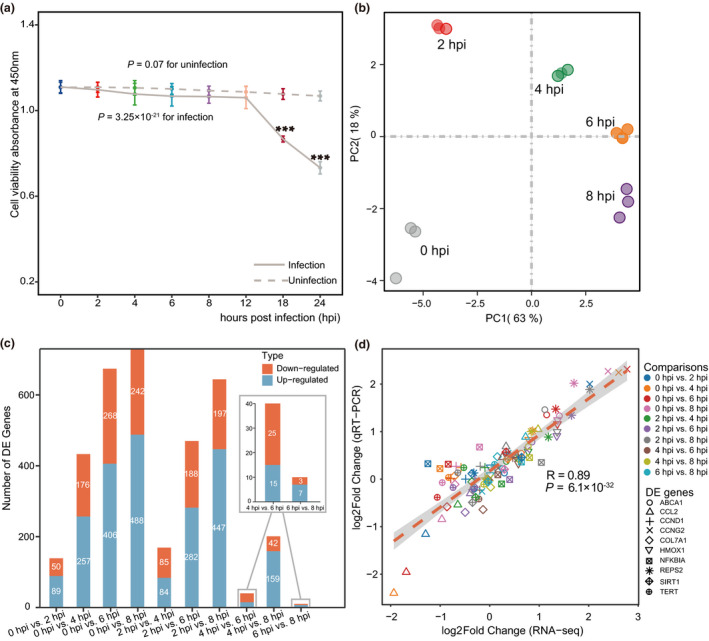
Genome‐wide gene expression analysis from host transcriptomes during pathogen infection.** (a)** Cell viability analysis used Cell Counting Kit‐8 at different times for alveolar epithelial cells (A549) after pneumococcal infection at a multiplicity of infection of 20 (20 pneumococci per epithelial cell). Solid line and dashed line show the viability of infected and mock‐infected epithelial cells, respectively. Data points represent the means ± s.d. obtained from six independent experiments with replicates. Statistical significance was assessed by one‐way ANOVA with *P* = 3.25 × 10^−21^ for infected cells and *P* = 0.07 for mock‐infected cells. ***Significant Tukey *post hoc* test when the values were compared with those of the control (*P* < 0.001). **(b)** Principal component analysis of all libraries based on detected genes in RNA‐sequencing (RNA‐seq). The first component explains 63% of the variability and the second component explains 18%. **(c)** Number of differentially expressed (DE) genes between libraries denoted by the histogram. The blue bars indicate the number of upregulated genes and the orange bars show the number of downregulated genes. **(d)** Gene expression correlation was validated between RNA‐seq and quantitative reverse transcription‐PCR (qRT‐PCR). The RNA samples were obtained from three independent experiments with replicates and each point represents the log_2_ fold change in DE genes in the two technologies. Each color represents a time point comparison from 10 comparisons. Each shape represents one of 10 validated DE genes (*HMOX1*, *REPS2*, *CCND1*, *NFKBIA*, *COL7A1*, *TERT*, *CCNG2*, *ABCA1*, *CCL2* and *SIRT1*). The correlation coefficients were calculated by Pearson’s test (*R* = 0.89, *P* = 6.1 × 10^−32^). hpi, hours postinfection.

To examine how cell viability changes during infection, we assessed the viability of alveolar epithelial carcinoma cell line (A549) exposed to *S. pneumoniae* (serotype 3) with a multiplicity of infection of 20 (i.e. 20 pneumococci per epithelial cell) at eight time points (0, 2, 4, 6, 8, 12, 18 and 24 hpi) using a Cell Counting Kit‐8 assay. We found that there was no significant difference in cell viability for mock‐infected A549 cells at all time points (*P* = 0.07, one‐way ANOVA test; Figure 1a). Although cell viability was different in the A549 cells over all time points postinfection (*P* = 3.25 × 10^–21^, one‐way ANOVA test), it was not significantly impaired at 2, 4, 6 and 8 hpi compared with that of control cells; however, cell viability was significantly reduced at 18 and 24 hpi (*P* = 2.64 × 10^‐12^ for 18 hpi; *P* = 4.63 × 10^‐13^ for 24 hpi, Figure 1a). Therefore, to further resolve the dynamic nature underlying host transcriptional responses to pathogen infection, we performed RNA‐seq on A549 cells to investigate global mRNA expression profiles in response to *S. pneumoniae* at the five time points (0, 2, 4, 6 and 8 hpi) that would eliminate the effect of reduced cell viability on host transcription levels.

To analyze the mRNA profiles in infected cells, we obtained an average of 26.76 million raw reads with lengths of 150 bp for each library from a total of 15 samples (three biological replicates for each time point). After removal of adapter sequences and low‐quality base trimming, we retained approximately 25.75 million clean reads per sample with clean bases ranging from 6.62 to 9.9 Gb. In general, at least 95.07% of reads for each sample were aligned to the human reference genome (Supplementary table 1). Principal component analysis showed that all samples were clearly separated into five groups with the same infection time for each group (Figure 1b). Using the DESeq2 package in R with expression cutoff values of > 1.5 fold change (FC) and false discovery rate (FDR) < 0.05, we identified 1330 DE genes in all comparisons during infection (Figure 1c, Supplementary table 2). To further validate these DE genes detected by RNA‐seq, we performed qRT‐PCR analyses by selecting 10 DE genes for all comparisons on independent RNA samples. A high correlation was observed between the FCs in expression obtained by qRT‐PCR and RNA‐seq [Pearson’s correlation (*R*) = 0.89, *P* = 6.1 × 10^−32^], revealing the consistency in gene expression between the two techniques (Figure 1d).

We also analyzed the contribution of DE genes to each time point in comparison with mock‐infected time (0 hpi). During this time course, a total of 1095 DE genes were induced in the four comparisons (Supplementary figure 1a). Of these, there were 139 DE genes in 2 hpi (45 unique genes), 433 in 4 hpi (103 unique genes), 674 in 6 hpi (133 unique genes) and 730 in 8 hpi (234 unique genes). In comparison with 0 hpi, there were 27 upregulated and 21 downregulated unique genes in 2 hpi, 48 upregulated and 55 downregulated unique genes in 4 hpi, 58 upregulated and 75 downregulated unique genes in 6 hpi and 162 upregulated and 74 downregulated unique genes in 8 hpi (Supplementary figure 1b, c).

### Cluster analysis of DE genes reflects biological processes and pathways essential to host–pathogen interactions

**Figure 2:**
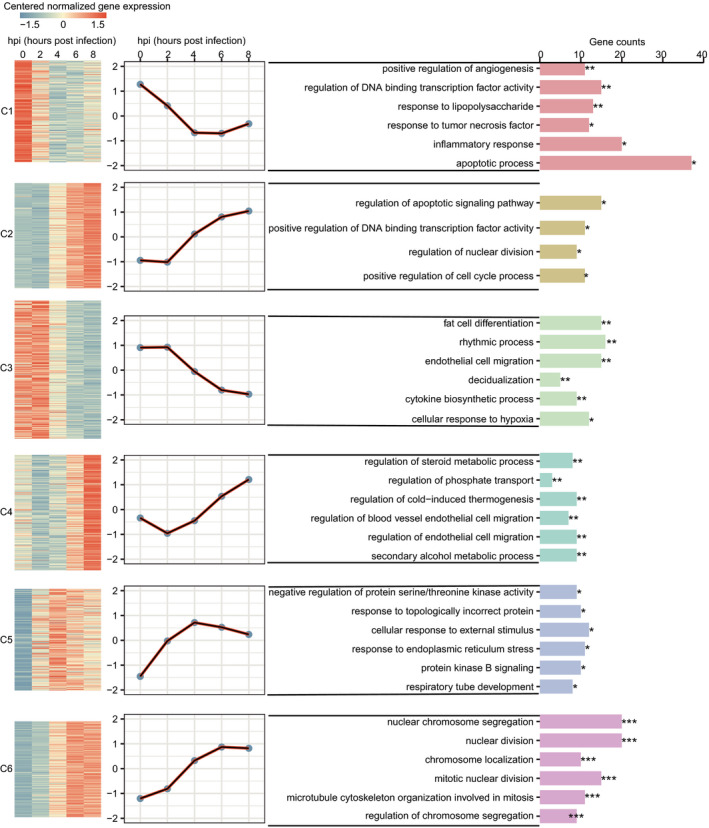
Clusters of genes showing representative expression patterns in epithelial cells during infection. The time‐series expression trends of differentially expressed (DE) genes were analyzed by R package Mfuzz, which can group DE genes into different clusters based on similar temporal expression patterns. The left heatmaps show clear clusters of coexpressed epithelial genes underlying host–pathogen interactions. Each line represents the normalized expression of one gene at different time points. The middle line charts show the expression trends of coexpressed epithelial genes along time series in each cluster, where the solid black lines represent the cluster centers. The right panel shows Gene Ontology (GO) annotation of each cluster performed by clusterProfiler package in R. The *P*‐values for enrichment were corrected for multiple testing using the false discovery rate (FDR). *FDR < 0.05, **FDR < 0.01 and ***FDR < 0.001. Gene counts represent the number of genes enriched in the GO functional term. hpi, hours postinfection.

To classify the dynamic changes in transcription of the 1330 DE genes, we implemented clustering analysis on temporal expression by adopting the Mfuzz package in R. All DE genes were separated into six optimal clusters based on the similarity of gene expression patterns over time, which often tended to be functionally related or participate in the same regulatory networks (Figure 2, Supplementary table 3). To gain further insights into the potential functions and biological characteristics of the DE genes in each cluster, we performed Gene Ontology (GO) term enrichment analysis and found several biological processes associated with inflammatory or immune responses (Figure 2). For instance, cluster 1 (C1) has a gradually decreasing trend of gene expression from 0 to 4 hpi, with significant enrichment in “response to tumor necrosis factor” and “inflammatory response” GO terms. The genes in C2 gradually increase in expression from 2 to 8 hpi and were enriched in “regulation of apoptotic signaling pathway.” Both “cytokine biosynthetic process” and “cellular response to external stimulus” were found in C3 and C5, respectively (Figure 2, Supplementary table 4). In addition, we found several biological processes associated with transcriptional regulation, such as “regulation of DNA‐binding transcription factor activity” in C1 and “positive regulation of DNA‐binding transcription factor activity” in C2 (Figure 2, Supplementary table 4). We also performed the GO analysis for DE genes contributing to each time point compared with 0 hpi and found several common biological processes associated with cellular response to external stimulus, regulation of DNA binding, cell apoptotic and transcriptional regulation in these later time points (Supplementary figure 2, Supplementary table 5). Moreover, several specific biological processes were also found for a certain time point, such as “response to oxidative stress” at 2 hpi and “intracellular receptor signaling pathway” at 4 and 6 hpi (Supplementary table 5).

**Figure 3:**
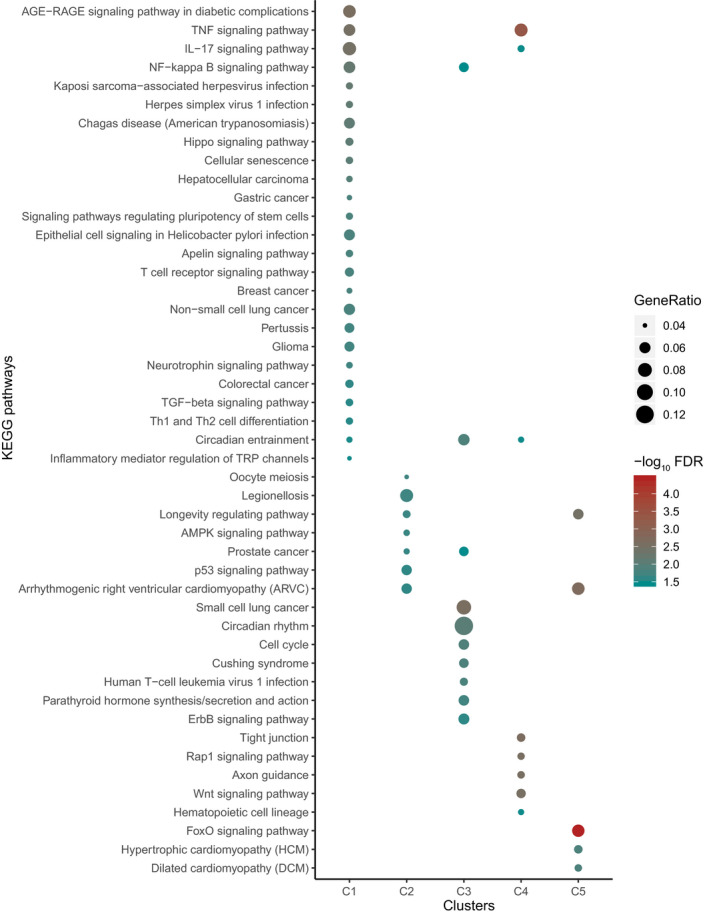
Kyoto Encyclopedia of Genes and Genomes (KEGG) pathway analysis for each cluster. The size of the nodes represents the value of the gene ratio, which indicates the number of enriched genes. The color shade of the nodes represents the *P*‐values corrected for multiple testing using the false discovery rate (FDR). IL, interleukin; NF‐kappa B, nuclear factor kappa B; TGF, tumor growth factor; Th1, T‐helper type 1; Th2, T‐helper type 2; TNF, tumor necrosis factor; TRP, transient receptor potential.

Moreover, to curate the important metabolic or signaling pathways from Kyoto Encyclopedia of Genes and Genomes (KEGG), we performed the KEGG pathway enrichment analysis of the DE genes in each cluster, and there were five clusters with significant enrichment (C1–C5). Among these five clusters, we also found several significantly targeted pathways (FDR < 0.05) involved in inflammatory or immune responses (Figure 3, Supplementary table 4). For instance, genes in C1 were enriched in the largest number of pathways, among which several pathways were relevant to inflammation and immune response, including the “tumor necrosis factor signaling pathway” and “IL‐17 signaling pathway,” which were also observed in C4 (Figure 3, Supplementary table 4). The majority of induced genes in these pathways encode several cytokines, chemokines or transcriptional regulators, such as CCL2, CXCL1, CXCL2, CXCL8, JUN, TRAF3 and NFKBIA, which have been determined to play a role in the immune response.^6^, ^14^, ^19^, ^20^, ^21^ In addition, the “NF‐kappa B signaling pathway” was also observed in both C1 and C3, which participate in the coordination of the inflammatory and immune responses through regulating the expression of hundreds of immune relevant genes, in particular those encoding proinflammatory cytokines and chemokines. Notably, we also found the “AMPK signaling pathway” specifically enriched in C2. The activation of AMPK by activators could inhibit inflammatory responses, indicating a role of AMPK signaling pathway in the depression of inflammation.^22^ In particular, we also found the “FoxO signaling pathway” in C5. FoxO signaling plays a vital role in an evolutionarily conserved mechanism of cross‐regulation of metabolism and innate immunity, and *foxo* mutants can be resistant to some infections.^23^ In addition, we examined the pathway associated with infection at each time point. KEGG enrichment analysis showed that several pathways relevant to inflammation and immune response were specifically enriched in a certain time point (Supplementary figure 3 and Supplementary table 5). For instance, several inflammation and immune response pathways including the “tumor necrosis factor signaling pathway,” “IL‐17 signaling pathway” and “B‐cell receptor signaling pathway,” were found to be induced 2 hpi. At 6 hpi, we found several pathways involved in kinase signaling and transcriptional activation, such as the “MAPK signaling pathway” and “NF‐kappa B signaling pathway.”

Given the crucial role of cell apoptosis and TFs in the regulation of inflammatory and immune responses to pathogen infection, we further investigated in detail the functional dynamic changes in relevant DE genes. We curated 155 DE genes potentially associated with apoptosis from the THANATOS database,^24^ 162 DE TFs from a previous study^10^ and 359 DE genes involved in innate immune response from the InnateDB database,^25^ which were provided in Supplementary table 6. By applying the fuzzy c‐means method, each category of genes was appropriately classified into three clusters, displaying complex expression patterns in response to *S. pneumoniae* infection (Supplementary figure 4).

### TF target‐enriched clusters are coregulated by potential key transcriptional regulators

**Figure 4:**
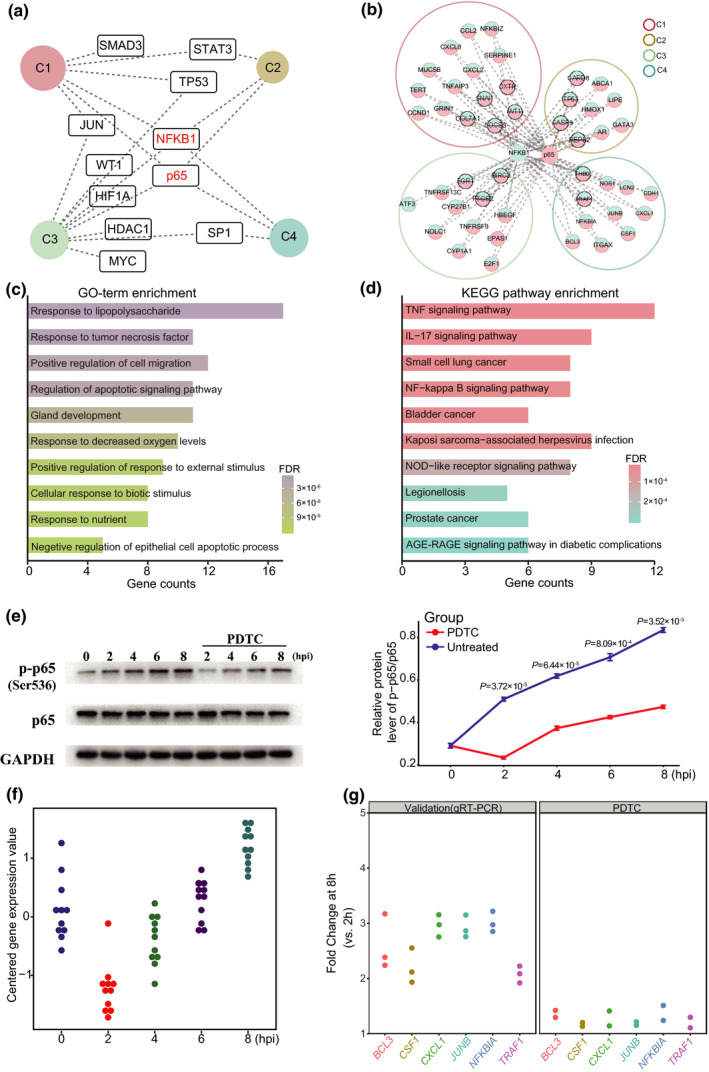
Transcription factors (TFs) and their targets in each cluster linked by transcriptional coregulation.** (a)** The TF target network of the differentially expressed (DE) genes in the different clusters. The dashed lines indicate enrichment in the clusters for TFs. **(b)** DE genes in four clusters coregulated by NFKB1 and p65. **(c and d)** The Gene Ontology (GO) and Kyoto Encyclopedia of Genes and Genomes (KEGG) enrichment analysis of the DE genes in four clusters coregulated by NFKB1 and p65. **(e)** A549 cells were coincubated with *Streptococcus pneumoniae* for five infection times. For pyrrolidine dithiocarbamate (PDTC) treatment, A549 cells were coincubated with *S. pneumoniae* and simultaneously treated with PDTC at 25 μm. Cell lysates were collected and immunoblotted with antibodies against p65 and p‐p65 (Ser^536^). Data are means ± s.d. from three independent experiments. Significant differences between groups with and without PDTC treatment were determined using a two‐tailed unpaired Student’s *t*‐test or one‐way analysis of variance. **(f)** The time‐series expression trends of DE genes regulated by p65 in cluster 4 (C4) enriched in the tumor necrosis factor (TNF) signaling pathway. **(g)** The fold change in DE genes regulated by p65 between 8 h postinfection (hpi) and 2 hpi in C4 upon treatment with PDTC. *N* = 3 independent experiments with replicates. FDR, false discovery rate; GAPDH, glyceraldehyde 3‐phosphate dehydrogenase; IL, interleukin; NF‐kappa B, nuclear factor kappa B; p‐p65, p65 phosphorylation.

TFs have been extensively demonstrated as important regulators in mediating host responses to pathogens.^8^, ^26^ We next explored the associations between the genes in the six clusters at the TF level based on target gene enrichment via the TRRUST database,^27^ and found 11 TFs that were predicted to link at least one of the four clusters (Figure 4a, Supplementary table 7). Among these TFs, there were six for C1, three for C2, nine for C3 and three for C4. Moreover, several TFs, such as NFKB1, p65, JUN, SP1 and STAT3, were demonstrated previously to be involved in the regulation of lung inflammation and immunity during pathogen infection or external stimulation.^1^, ^7^, ^8^ Meanwhile, we also found that both NFKB1 and p65 were significantly enriched for target genes in four of the clusters (Figure 4a). Among these target genes, 45/47 genes were coregulated by *NFKB1* and *p65* (Figure 4b).

Both NFKB1 and p65 are important members of the nuclear factor‐kappa B (NF‐κB) family, which are recognized as essential regulators in inflammatory processes and innate immunity, as well as in modulation of the transcriptional activity of NF‐κB.^7^, ^28^ GO analysis showed that these target genes were significantly enriched in several biological processes associated with host responses to pathogen infection or external stimuli, such as “response to tumor necrosis factor,” “regulation of apoptotic signaling pathway” and “positive regulation of response to external stimulus” (Figure 4c). Furthermore, KEGG analysis of these target genes revealed several critical pathways associated with host responses against pathogens, such as the “tumor necrosis factor signaling pathway,” “IL‐17 signaling pathway” and “NF‐κB signaling pathway” (Figure 4d). These analyses further confirmed the role of both NFKB1 and p65 in the regulation of immune system responses and NF‐κB activation pathway. GO and KEGG enrichment analyses for NFKB1 and p65 target genes in each cluster also showed similar results to those in all clusters (Supplementary figure 5).

In addition, we attempted to investigate the phosphorylation level of p65 at different times during infection. Although there were no significant changes in p65 expression at the mRNA and protein levels during *S. pneumoniae* infection, the phosphorylation of p65 in A549 cells gradually increased in a time‐dependent manner in response to infection (Figure 4e). To further validate the regulatory role of p65 phosphorylation (p‐p65) toward potential target genes, we utilized the NF‐κB inhibitor [pyrrolidine dithiocarbamate (PDTC)] to treat the A549 cells during *S. pneumoniae* infection and found that the level of p‐p65 was obviously decreased at any infection time with PDTC treatment than without treatment (Figure 4e). Similarly, the reduced levels of p‐p65 during *S. pneumoniae* infection were also observed in both nuclear and cytosolic fractions of A549 cells with PDTC treatment (Supplementary figure 6). We selected the DE genes (including *BCL3*, *CSF1*, *CXCL1*, *JUNB*, *NFKB1A* and *TRAF1*) coregulated by NFKB1 and p65 that were enriched in the tumor necrosis factor signaling pathway belonging to C4. These DE genes at 8 hpi showed increased expression compared with 0 hpi, but more significantly increased expression than 2 hpi (all *P*‐values < 0.05, Figure 4f). Upon treatment with PDTC, we found significant reductions in the FCs of these genes between 8 and 2 hpi compared with those determined by both RNA‐seq and qRT‐PCR without PDTC treatment (all *P*‐values < 0.05, Figure 4g).

### Dynamic changes in miRNAs at different infection time points

**Figure 5:**
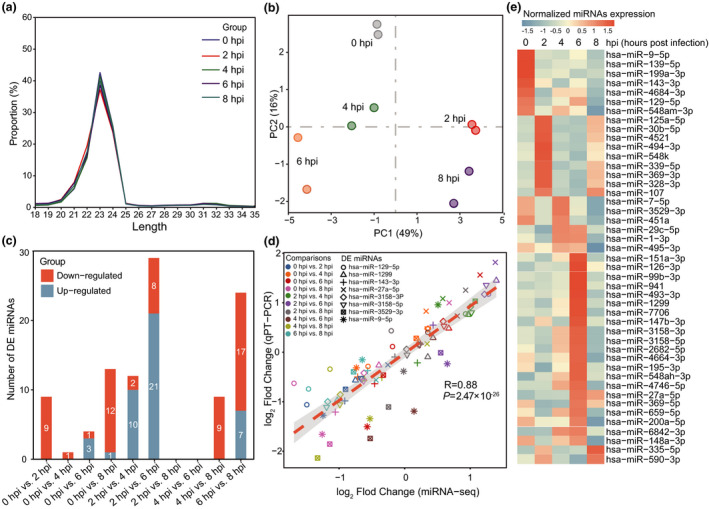
Dynamic changes in microRNAs (miRNAs) from miRNA‐sequencing (miRNA‐seq) in host cells during infection. **(a)** The length distribution of small RNAs at different infection time points. The *x* axis represents the small RNA length, ranging from 18 to 35 nucleotides, and the *y* axis represents the ratio of small RNAs of different lengths to the total number of small RNAs. **(b)** Principal component analysis for miRNA expression of the five periods in all libraries. **(c)** The histogram graph shows the number of differentially expressed (DE) miRNAs between any pair of infection stages. The white numbers on the blue bars indicate the number of upregulated genes and the orange bars show the number of downregulated genes. Both false discover rate < 0.05 and fold change >2 were set as the threshold for DE miRNAs. **(d)** Pearson’s test was used to analyze the correlation between miRNA‐seq and quantitative reverse transcription‐PCR (qRT‐PCR) for DE miRNAs (*R* = 0.88, *P* = 2.47 × 10^−26^). The RNA samples were obtained from three independent experiments with replicates. Each color represents a time point comparison from 10 comparisons. Each shape represents one of eight validated DE miRNAs (hsa‐miR‐3158‐3P, hsa‐miR‐1299, hsa‐miR‐143‐3p, hsa‐miR‐3158‐5p, hsa‐miR‐3529‐3p, hsa‐miR‐9‐5p, hsa‐miR‐27a‐5p and hsa‐miR‐129‐5p). **(e)** Heatmap of the 45 DE miRNAs at different stages postinfection. In each of these cells, orange represents the highest expression, and blue represents the lowest expression. hpi, hours postinfection.

To investigate the dynamic change in miRNA expression during infection, small RNA libraries paired with mRNA libraries were prepared for miRNA‐seq. However, because of the failure of library construction and/or sequencing, we finally obtained high‐quality miRNA‐seq data sets from two biological replicates for each time point. After filtering out low‐quality reads, trimming off adaptors and removing RNA fragments less than 18 nucleotides, we retained an average of 6 713 283 clean reads per sample, with an average mapping rate of 94.27% for small RNAs (Supplementary table 8). Of these high‐quality small RNAs, the length distribution was similar throughout all time points with approximately 90% of the small RNAs ranging from 21 to 24 bp in length (Figure 5a). In total, we identified an average of 3 346 306 reads as known miRNA sequences by aligning the reads to human miRNA reference sequences in miRbase version 21 (Supplementary table 8). The principal component analysis shows the distribution of all samples in the form of a two‐dimensional scatter diagram, indicating significant discrimination for the overall expression patterns of miRNAs at each time point (Figure 5b).

We next performed differential expression analysis of miRNAs using the DESeq2 tool with thresholds of a FC > 2 and FDR < 0.05, and obtained 45 robust DE miRNAs in all comparisons during infection (Figure 5c). In contrast to the differential expression of mRNAs, there were more downregulated miRNAs than upregulated miRNAs in most comparisons, suggesting the role of miRNAs in the negative regulation of their target mRNAs by either translational repression or mRNA degradation (Figure 5c, Supplementary table 9). For the contribution of DE miRNAs to each time point in comparison with 0 hpi, we found that there was a total of 19 DE miRNAs in the four comparisons. Of these, there were nine DE miRNAs in 2 hpi (three unique miRNAs), one in 4 hpi, four in 6 hpi (three unique miRNAs) and 13 in 8 hpi with seven unique miRNAs (Supplementary figure 7). To further evaluate the miRNA‐seq data, we performed qRT‐PCR analyses on eight miRNAs selected in all comparisons using independent RNA samples. As a result, a relatively high correlation was observed between the FCs obtained by qRT‐PCR and miRNA‐seq: *R* = 0.88 and *P* = 2.47 × 10^−26^ (Figure 5d). Among these 45 DE miRNAs, there were seven with peak expression at 0 hpi, followed by nine at 2 hpi, six at 4 hpi, 21 at 6 hpi and two at 8 hpi (Figure 5e).

### Integrative analysis of miRNA and mRNA expression reveals the miRNA–mRNA networks associated with host–pathogen interactions

**Figure 6:**
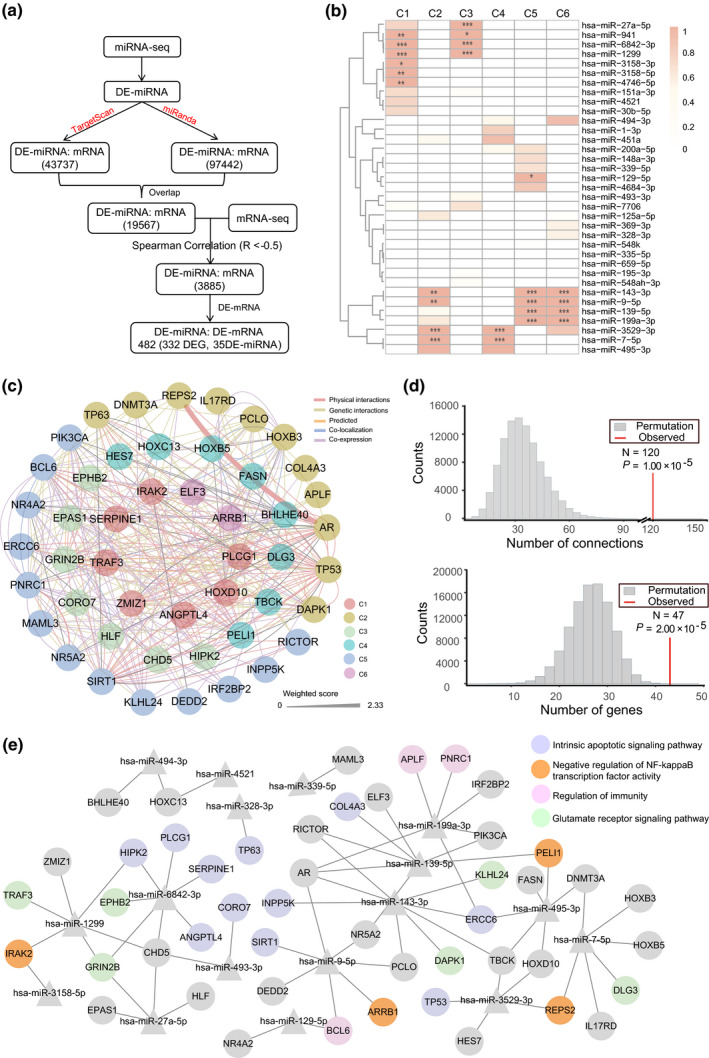
Identification of miRNA–mRNA regulatory networks associated with host–pathogen interactions.** (a)** Flowchart for detecting potential interactions between differentially expressed (DE) miRNAs and DE mRNAs acting in A549 cells. DE miRNA indicates DE miRNA, and DE mRNA indicates DE mRNA. **(b)** The heatmap shows the false discovery rate (FDR; hypergeometric test) for enrichment of DE miRNA target genes in each cluster after correction for multiple comparisons. The intensity of color in each rectangle explains the enrichment degree of genes targeted by DE miRNA in each cluster. *FDR < 0.05; **FDR < 0.01; ***FDR < 0.001. **(c)** Module 1 (M1) with the highest score of 6.791 was obtained from a huge complex biological network by MCODE plug‐in of Cytoscape, comparing 47 nodes and 120 edges. The colors of connections represent different interaction types. The filled color of each node represents the cluster to which the DE gene was assigned. **(d)** A permutation test for interconnection among M1 based on the STRING database. The histograms show the distribution of genes (lower plot) and connections (upper plot) of 1 000 000 iterations from STRING, and the vertical red lines show the real values of number of genes and connections in the network of M1. The observed numbers and empirical *P*‐values are shown in each plot. **(e)** The regulatory network relationship between miRNAs and target genes in M1. The triangle nodes represent miRNAs. The filled color of each circle node represents the corresponding biological process in which the node gene might be involved. miRNA, microRNA; mRNA, messenger RNA.

In general, miRNAs negatively regulate the expression of their target mRNAs by silencing or degradation.^15^ Considering the anticorrelated relationship between miRNA and mRNA, we attempted to identify putative miRNA–mRNA regulatory interactions for further understanding of the host transcriptional response. From 19 567 shared DE miRNA–mRNA pairs by TargetScan and miRanda predictions, we recognized 3885 DE miRNA–mRNA pairs with anticorrelation analysis (*R* < −0.5), from which 482 DE miRNA–DE mRNA pairs were retained, consisting of 332 DE mRNAs and 35 DE miRNAs (Figure 6a, Supplementary table 10). We adopted enrichment analysis with hypergeometric tests to quantitatively assess the effects of the DE miRNAs on 332 DE genes during infection, and found that 272 DE genes targeted by 14 DE miRNAs were highly enriched in different clusters (Figure 6b, Supplementary table 11). For example, the target genes of three miRNAs (hsa‐miR‐941, hsa‐miR‐6842‐3p and hsa‐miR‐1299) were mainly enriched in both C1 and C3. For hsa‐miR‐143‐3p and hsa‐miR‐9‐5p, their target genes showed significant enrichment in C2, C5 and C6 (Figure 6b). Moreover, the comparison of the time‐series expression trends of these 14 DE miRNAs and their target mRNAs showed that these target mRNAs were obviously negatively modulated by the DE miRNAs in the enriched clusters (Supplementary figure 8).

To gain insight into the biological function and interconnectedness of these 332 DE genes targeted by DE miRNAs, we constructed an interconnected biological network with 279 nodes and 3700 edges based on the five types of biological relationships of these genes from GeneMANIA (Supplementary figure 9). To further find the functional module (highly interconnected regions) from the complex biological network, we used the MCODE tool to analyze the corresponding networks with the cutoff criteria of an MCODE score > 5 and a degree cutoff > 3, and identified the two most densely interconnected modules: module 1 (M1) and module 2 (M2) (Figure 6c, Supplementary figure 10a). Moreover, to further evaluate the reality and significance of the observed modules, we compared the number of nodes/genes and their edges/connections in physical interactions from two modules with those of 100 000 random iterations from the STRING database. The permutation test analyses for M1 showed significantly more nodes/genes (*P* = 2 × 10^−5^) and edges/connections (*P* = 1 × 10^−5^) than random expectations (Figure 6d), and statistical significance for the number of genes (*P* = 2 × 10^−5^) and connections (*P* = 2 × 10^−5^) was also observed in M2 (Supplementary figure 10b). In addition, most of the DE genes in M1 were assigned to C2 and C5 (24 of 47). GO enrichment analysis revealed that the DE genes in M1 are mainly associated with the “negative regulation of NF‐kappaB transcription factor pathway,” “regulation of immunity,” “cell apoptotic process,” and “glutamate receptor signaling pathway” (Supplementary figure 11, Supplementary table 12). However, no significant biological process enrichment for DE genes was observed in M2.

Given the complex relationships between miRNAs and their target genes, with one gene potentially regulated by multiple miRNAs or multiple genes potentially regulated by one miRNA, we performed network analysis to investigate the regulatory relationships between DE miRNAs and target DE genes in M1 and M2. The two networks consisted of 48 DE genes regulated by 17 DE miRNAs for M1 and 40 DE genes regulated by 18 DE miRNAs for M2 (Figure 6e, Supplementary figure 10c). For M1, we found that *BCL6* targeted by hsa‐miR‐129‐5p and miR‐9‐5p, and *APLF* and *PNRC1* targeted by hsa‐miR‐199a‐3p are associated with regulation of immunity. Remarkably, there were 11 DE genes related to apoptotic signaling pathways regulated by 10 DE miRNAs, in which several miRNAs were found to target genes involved in the negative regulation of NF‐κB TF activity, such as hsa‐miR‐1299, hsa‐miR‐3158‐5p, hsa‐miR‐9‐5p, hsa‐miR‐139‐5p and hsa‐miR‐495‐3p.

## Discussion

Lung epithelial cells contribute to the first line of host defense against pathogen infections, often acting as important mediators in the initiation of immune responses by inducing the production of various cytokines and chemokines for pathogen clearance.^1^, ^2^ Pathogen infection in host cells is a complex multifactorial interaction, which leads to abundant gene expression changes involved in disease processes.^1^, ^9^ RNA‐seq studies investigating the global gene expression changes for epithelial infection of *S. pneumoniae* using various cell lines have been performed.^4^, ^11^ Compared with these previous RNA‐seq studies—especially, the time‐resolved dual RNA‐seq analyses by Aprianto *et al*.^11^—our *in vitro* transcriptomic analyses first used serotype 3 *S. pneumoniae* for A549 infection, given that the strain tend to cause severe complications of invasive pneumococcal disease.^17^, ^18^ In addition, we applied TF‐binding site enrichment analysis and identified several key TFs, including common TFs observed in previous studies (NFKB1 and p65) as well as several specific TFs in our studies, such as JUN and SP1. Furthermore, we used miRNA‐seq data to monitor the miRNA expression changes in a time‐resolved manner that was not reported in previous RNA‐seq studies. Finally, our integrated analyses of mRNA‐seq and miRNA‐seq identified several candidate DE miRNAs associated with host inflammatory and immune responses, such as miR‐9, miR‐27 and miR‐199. The application of time‐resolved miRNA–mRNA integrated analyses might provide a comprehensive molecular basis underlying host cell responses to pathogen infections.^29^

Interplay between various pneumococcal strains and the epithelium often triggered the strain‐dependent host transcriptomic response.^4^, ^14^, ^30^ These can be explained partly by difference in genetic background or microinvasion between these strains with diverse capsular serotypes, such as D39 (serotype 2), G54 (serotype 19F), TIGR4 (serotype 4), BHN 418 (serotype 6B) and P1121 (serotype 23F).^4^, ^14^ In comparison with mock‐infected epithelial cells, infection with serotype 3 strain upregulated 89 genes at 2 hpi and 406 genes at 6 hpi, which was comparable to that observed with D39 or 23F but more than that observed with 6B.^4^, ^14^ Similarly, pathway enrichment analysis for the DE genes between 6 and 0 hpi also showed a broader number of biological pathways than the 6B strain, consistent with TIGR4 and 23F strains (Supplementary table 5).^4^, ^14^ Although our GO and KEGG analyses also showed several common inflammatory and immune response pathways induced by serotype 3 strain as well as other serotype strains, we found several specific biological processes or signaling pathways based on cluster analysis, such as “the AMPK signaling pathway” in C2, “negative regulation of calcium ion transport” in C4 and “response to endoplasmic reticulum stress” in C5. The activation of the AMPK signaling pathway had a prominent effect in the depression of inflammation.^22^ Previous studies have suggested that Ca^2+^ fluxes in epithelial cells are activated by pathogen contact and induce local cytokine and mucin secretion.^31^, ^32^, ^33^ Pulmonary pathogens also cause endoplasmic reticulum stress as a result of the accumulation of unfolded proteins, which leads to proinflammatory cytokine production in lung epithelial cells.^34^

Consistent with the enrichment analysis of DE genes in each cluster, several biological processes and KEGG pathways on each time point also showed the relevance to cell apoptotic and immune responses. However, several specific enrichment for biological processes and pathways was also observed at a certain time point. Many epithelial glutathione‐associated genes could be activated by reactive oxygen species produced by *S. pneumoniae*, indicating that the oxidative stress may be involved in the host–pathogen interactions.^11^ In addition, several components of the MAPK signaling pathways had been induced by *S. pneumoniae* strains with different serotypes and reported to play pivotal roles in cell growth, inflammation and apoptosis.^14^

The role of extensive TFs identified in the genome of Eukarya, Bacteria and Archaea has been elucidated.^10^, ^35^, ^36^ Considering the key regulatory role of TFs in determining the host response to pathogenic stimuli,^10^ we performed TF‐binding site enrichment analysis and found that both NFKB1 (processed to p50) and p65 (also called RELA) had target gene enrichment in all clusters. Both TFs belong to the NF‐κB family, which comprises a group of key regulators involved in diverse cellular processes associated with inflammatory and immune responses against pathogen infections.^7^, ^28^ The role of these two TFs in infection has been demonstrated by the host cells in response to the adherence of *S. pneumoniae* strains with different serotypes *in vitro*, including 6B, 23F and TIGR4.^4^ Extracellular stimulations induce the phosphorylation of IκB family members, which undergo proteasomal degradation, and lead to the nuclear translocation of heterodimeric p50/p65 that finally modulates gene expression.^7^ Moreover, previous studies have established the role of p‐p65 in the nuclear translocation of p65 and enhancement of the transcriptional activity of NF‐κB in human epithelial cells following exposure to environmental stimuli.^37^, ^38^ Treatment of human lung epithelial cells with thrombin was previously shown to increase p‐p65 at Ser^276^, which ultimately led to NF‐κB activation and IL‐8/CXCL8 release.^37^ Similarly, farnesol‐induced phosphorylation of p65 at Ser^276^ was shown to increase the transcriptional activity of NF‐κB and facilitate the expression of a number of genes mediating immune and inflammatory responses in human lung epithelial cells.^38^

In contrast to the 6B strain, serotype 3 strain induced considerable enrichment of more diverse TFs with 11 TFs predicted to link at least one of four clusters, similar to the TIGR4 and 23F strains.^4^ Besides commonly enriched binding sites for p50 and p65 among these diverse strains, the responses to serotype 3 revealed the enriched binding sites for STAT3 in C1 and C2, which was also observed in response to the TIGR4 strain.^4^ Recent studies have demonstrated the role of STAT3 in the control of lung inflammation and immunity.^6^, ^39^ Moreover, our analysis revealed particular enrichment of binding sites for TFs such as JUN and SP1. As a component of activator protein‐1, JUN showed binding site enrichment in C1 and C3, and is known to induce gene expression involved in mucus biosynthesis and secretion, including *MUC5AC* and *MUC5B*, from respiratory epithelial cells in response to bacterial stimuli,^40^, ^41^, ^42^ consistent with the GO term enrichment for DE genes in C3. In addition, the induction of mucins such as MUC5AC and MUC5B by stimulants was shown previously to lead to the activation of the TF SP1,^8^, ^43^, ^44^, ^45^ which we revealed to have target gene enrichment in C3 and C4. These analyses of the regulatory mechanisms of epithelial cells exposed to external stimuli suggest the involvement of these identified TFs in the inflammatory and immune responses to infections.

There has been an accumulating body of evidence supporting the regulatory role of miRNAs in the modulation of host–pathogen interactions.^15^, ^46^ In our study, module analyses showed two important modules from the biological network constructed by 332 DE genes negatively regulated by 35 DE miRNAs. We also found that the top one (M1) showed enrichment in several processes associated with immunity, apoptosis and transcriptional regulation of NF‐κB, consistent with previous studies.^15^ For instance, Sirtuin 1, the prototypic class III histone deacetylase, had been reported to function in the induction of immune and defense genes in pulmonary epithelial cells by *S. pneumoniae*, and pharmacologic activation of Sirtuin 1 might indicate a novel treatment strategy for bacterial infection.^47^, ^48^ The knockout of *TRAF3* in mice showed that *TRAF3* could regulate immune responses in myeloid cells and act to inhibit inflammation in mice,^21^ although its role in the epithelial cells remains unknown. These findings also highlight several components of the biological subnetworks which are targeted as part of the pneumococcal evasion strategies. Moreover, several candidate key DE miRNAs regulating DE gene targets in M1, such as miR‐9,^49^, ^50^ miR‐27^51^ and miR‐199,^52^ have been reported to be involved in inflammatory and immune responses during infections. However, it should be noted that each mRNA related to immunity and apoptosis in M1 is potentially regulated by multiple miRNAs or a single miRNA may target multiple genes (Figure 6), indicating the possibility of coordinated regulation of multiple targets by multiple miRNAs.^16^ Considering the complexity of miRNA–mRNA interactions, it is also necessary to validate each potential regulatory miRNA employing genetic modification of miRNAs in cell or animal models, which will aid in refining our understanding of these complicated systems.^15^, ^16^

Nevertheless, there are some limitations to our study. One limitation is the limited biological replicates per time point for RNA‐seq and miRNA‐seq, although to ensure consistency and robustness of positive findings, we performed qRT‐PCR replication for selected gene expression. It might still neglect several DE genes or miRNAs that contribute to host response to infection, thus more replicates are needed to fully characterize the expression changes associated with host–pathogen interactions. Our infection model relied on only one pneumococcal serotype strain and one type of cell line, leading to our findings limited by direct comparisons with multiple pneumococcal serotype strains and types of epithelial cell lines or primary cells. It is well‐known that different pneumococcal strains often cause distinct host response because of diverse capsular serotypes.^4^, ^14^ Similarly, cell origin and type may cause differences in the abilities of *S. pneumoniae* adhesion and invasion.^5^ It is therefore necessary to further explore the transcriptome of multiple types of epithelial cells during infection using different pneumococcal serotype strains. Furthermore, many of the DE miRNAs found in this study need to be further investigated to determine their biological function and likely regulated role in infection process.

In conclusion, we have described comprehensive transcriptional changes in human lung alveolar epithelial cells in a time‐resolved manner using RNA‐seq and miRNA‐seq. Of particular note, these data sets have been successfully used to identify several crucial biological processes and key regulators associated with inflammatory and immune responses based on bioinformatics or function‐related analyses, providing more biological insight into the pathogenesis of infection. Finally, our integrative analysis of the regulatory interactions between DE miRNAs and DE mRNAs might help us to comprehensively understand the molecular basis underlying host cell responses to pathogen infections.

## Methods

### Culture of the epithelial cell line and *S. pneumoniae*

The A549 cell was purchased from the Cell Resource Center, Shanghai Institutes for Biological Sciences of Chinese Academy of Sciences, and cultivated in Dulbecco’s modified Eagle’s medium (Gibco, Waltham, MA, USA) containing 10% fetal bovine serum (Gibco), penicillin (100 U mL^−1^) and streptomycin (100 mg mL^−1^) at 37°C in a humidified incubator with 5% CO_2_. Twenty‐four hours before the experiments, the cells were grown in medium without antibiotic supplements. *S. pneumoniae* serotype 3 strain (ATCC 6303) was purchased from American Type Culture Collection (ATCC, Manassas, VA, USA). Single‐colony isolates of *S. pneumoniae* were grown overnight on blood agar plates at 37°C with 5% CO_2_.

### Infection studies and measurement of cell viability

For infection of A549 cells, single colonies were expanded by resuspension in brain heart infusion broth and incubation at 37°C until the midlog phase (OD_600_, 0.3–0.4), and then were harvested by centrifugation. A549 cells were inoculated with *S. pneumoniae* resuspended in cell culture medium without antibiotics at 37°C and 5% CO_2_. For each experiment, a new aliquot of bacteria was slowly thawed and added to the cell medium at a multiplicity of infection of 20 (i.e. 20 pneumococci per epithelial cell). All experiments were performed in triplicate.

A549 cells were seeded at a density of 5 × 10^4^ cells per well (100 μL) in 96‐well plates. At 70–80% confluence, the cells were incubated with *S. pneumoniae* at 37°C for the indicated times. Cell viability was measured using Cell Counting Kit‐8 (Dojindo Molecular Technologies, Kumamoto, Japan), following the manufacturer’s instructions. The absorbance was measured in a multifunction microplate reader (BioTek, Winooski, VT, USA) at 450 nm.

### RNA extraction, library construction and sequencing

Total RNA was isolated from A549 cells after pneumococcal infection according to standard procedures using TRIzol Reagent (Thermo Fisher Scientific, Waltham, MA, USA). The purity and quality of the RNA were examined using a Nano Photometer spectrophotometer (Implen, Munich, German) and the Agilent Bioanalyzer 2100 system (Agilent Technologies, Santa Clara, CA, USA); RNA samples having an RNA integrity number > 7.0 were used for further analysis. About 5 μg of total RNA per sample (with three biological replicates) was used as input material for complementary DNA (cDNA) library construction using an Illumina TruSeq RNA Sample Preparation Kit (Illumina, San Diego, CA, USA). Approximately 3 μg of total RNA from each sample (with three biological replicates) was used to prepare a small RNA library generated using the NEBNext Multiplex Small RNA Library Prep Set for Illumina (NEB, Ipswich, MA, USA), following the manufacturer’s protocol. The quality of each cDNA library and small RNA library was assessed on the Agilent Bioanalyzer 2100 system. The cDNA and small RNA libraries were sequenced on the Illumina HiSeq 4000 platform with 150‐bp paired‐end reads generated.

### Data preprocessing

RNA‐seq raw reads were processed in a similar manner as previously described.^53^, ^54^ Both 3′ and 5′ adapter contaminants were removed by Cutadapt, and the full‐length reads were processed as FASTA format using an in‐house pipeline.^55^ After preprocessing, the quality of these reads was evaluated by FastQC and further visualized by SplicingViewer software.^56^ Read alignments to the human genome and count detections were conducted using Hiast2 and StringTie, respectively.

For the small RNA library sequencing data, high‐quality trimmed reads were obtained after quality control, including removal of low‐quality reads from the raw data and reads with adapter contaminants. The remaining reads with lengths ranging from 18 to 35 nucleotides were chosen for further analysis. The clean data were mapped to the hg38 reference sequence and aligned against miRbase version 21 by Bowtie.^57^

### Differential expression analysis and validation of DE genes or miRNAs by qRT‐PCR

DE genes and DE miRNAs in cells infected by bacteria between any two points were identified by the DESeq2 package in R^58^ based on the raw counts obtained from the previous steps. The *P*‐values for differential expression analysis were corrected by the Benjamini and Hochberg FDR procedure in DESeq2. For the RNA‐seq data, both FDR < 0.05 and FC > 1.5 were set as the thresholds for DE genes. For the miRNA‐seq data, both FDR < 0.05 and FC > 2 were set as the thresholds for DE miRNAs.

The selected DE genes were validated by qRT‐PCR to confirm the robustness of RNA‐seq. Each cDNA was synthesized from 1 μg of total RNA using the GoTaq 2‐Step qPCR System Kit (Promega, Madison, Wisconsin, USA). qRT‐PCR was performed on the Applied Biosystems QuantStudio Real‐time PCR Instrument (Thermo Fisher Scientific), according to the manufacturer’s protocols. All measurements for each sample were performed in triplicate, and the FC in a gene was calculated based on the 2^–△△C^ *^t^* method after normalization against β‐actin. All relevant primers are listed in Supplementary table 13. The correlation between RNA‐seq FCs and FCs from qRT‐PCR was calculated using Pearson’s test.

For the validation of expression data generated by miRNA‐seq, each cDNA was synthesized from 1 μg of RNA by the miScript II RT Kit (Qiagen, Dusseldorf, Germany). qRT‐PCR was performed for each sample in triplicate with U6 as an internal control. The qRT‐PCR instrument and the calculation of FCs in miRNAs were identical to those described previously. The Bulge‐loop miRNA qRT‐PCR primer sets (one RT primer and a pair of qPCR primers for each set) specific for eight miRNAs were designed and synthesized by RiboBio (Guangzhou, China). The correlation between miRNA‐seq FCs and FCs from qRT‐PCR was calculated by Pearson’s test.

### Time‐series clustering of DE gene enrichment

We clustered DE genes along a time series using Mfuzz (version 2.42.0) in the R package based on the fuzzy c‐means method.^59^ Log transformations of the average expression value of each gene at each time point were processed to construct an ExpressionSet object and standardize the expression values. The standardized expression data were used as input to generate the clusters with membership values over 0.5 for each DE gene and at least 90% of the DE genes were assigned to a unique cluster.

### Key regulators for DE genes in each cluster

We determined whether DE genes in all clusters were regulated by TFs based on predictions using the TRRUST database.^27^ A transcriptional regulatory relationship between a TF and DE genes in each cluster was considered statistically significant with the following thresholds: a corrected *P* value < 0.05 and gene counts > 5. The TF target networks were constructed by Cytoscape version 3.4.0.^60^

### Protein extraction, western blot assay and drug treatment

Total protein was extracted from cultured cells at different time points using a protein extraction kit (KeyGen Biotech, Nanjing, China). After infection, the cultured cells were simultaneously treated with the NF‐κB inhibitor PDTC (Sigma Chemical Company, St. Louis, MO, USA) at 25 μm for the different time intervals to inhibit p‐p65. An equal amount of extracted protein was separated by sodium dodecyl sulfate–polyacrylamide gel electrophoresis and transferred to a polyvinylidene difluoride membrane. After blocking with 5% skim milk in Tris‐buffered saline, 0.1% Tween 20 (TBST) for 1 h at room temperature, the membrane was incubated with antibodies against p65 (Catalog number: 8242; Cell Signaling Technology, Boston, MA, USA) or p‐p65 at Ser^536^ (Catalog number: 3033; Cell Signaling Technology). Antibodies against glyceraldehyde 3‐phosphate dehydrogenase (Catalog number: 10494‐1‐AP; Proteintech, Rosemont, USA) and proliferating cell nuclear antigen (Catalog number: 10205‐2‐AP; Proteintech, Rosemont, USA) were used as endogenous control for nucleus and cytosol, respectively. Image Lab version 5.2 software was used to calculate the relative expression levels of each protein. Data were presented as the means ± s.d. from three independent experiments. Significant differences between groups were determined using a two‐tailed unpaired Student’s *t*‐test or one‐way analysis of variance. Cytoplasmic and nuclear fractionation from A549 cells was performed according to standard procedures using Nuclear and Cytoplasmic Extraction Reagents (catalog number: 78833; Thermo Fisher Scientific).

### miRNA target prediction and correlation analysis

To identify potential interactions of miRNAs with their target mRNAs, we predicted the target DE miRNA–mRNA pairs using TargetScan^61^ and the miRanda database.^62^ DE miRNA–mRNA pairs detected simultaneously in both data sets were used for subsequent analysis. We next calculated Pearson’s correlation coefficient (*R*) between expression levels of each DE miRNA and its predicted mRNAs, in which only those DE miRNA–mRNA pairs with *R* < −0.5 were chosen. Then, we extracted the DE miRNA–DE mRNA pairs based on the DE mRNAs identified in this study.

### Network construction using MCODE and permutation analysis

The interactive network for all DE genes was built using the GeneMANIA software based on physical interactions, genetic interactions, predicted functional relationships, colocalization and coexpression.^63^ Then, we employed the MCODE plugin in Cytoscape to detect higher connected modules in the network with scores > 5.^64^ By adopting a permutation test with 100 000 iterations based on the ranking of genes and their connections, we assessed statistical significance for the number of interacting genes and connections relative to random expectations. All the networks were visualized using Cytoscape version 3.4.0.

### Enrichment analysis using Gene Ontology terms and Kyoto Encyclopedia of Genes and Genomes pathways

In this study, GO analysis was used to annotate genes and gene products, and dissect the biological characteristics of associated gene set.^65^ The KEGG analysis was used to analyze functional and metabolic pathways from the associated gene set.^66^ GO enrichment analysis in biological process and KEGG were used to annotate genes by the “enrichGO” and “enrichKEGG” function of the clusterProfiler^67^ package in R, respectively. GO terms and KEGG pathways with corrected *P*‐values (FDR) < 0.05 were considered to be meaningful enrichments.

## Conflict of Interest

The authors declare no conflict of interest.
