# Supplementary material for: Time‐resolved mRNA and miRNA expression profiling reveals crucial coregulation of molecular pathways involved in epithelial–pneumococcal interactions
Source: Immunol Cell Biol. 2020 Jul 20;98(9):726–42. doi: 10.1111/imcb.12371 (PMC7586809; doi:10.1111/imcb.12371)
Supplement: Supplementary file 1 [file IMCB-98-726-s001.zip › imcb12371-sup-0013-Supinfo.pdf]

Supplementary Material

Supplementary figures and tables

Supplementary figures

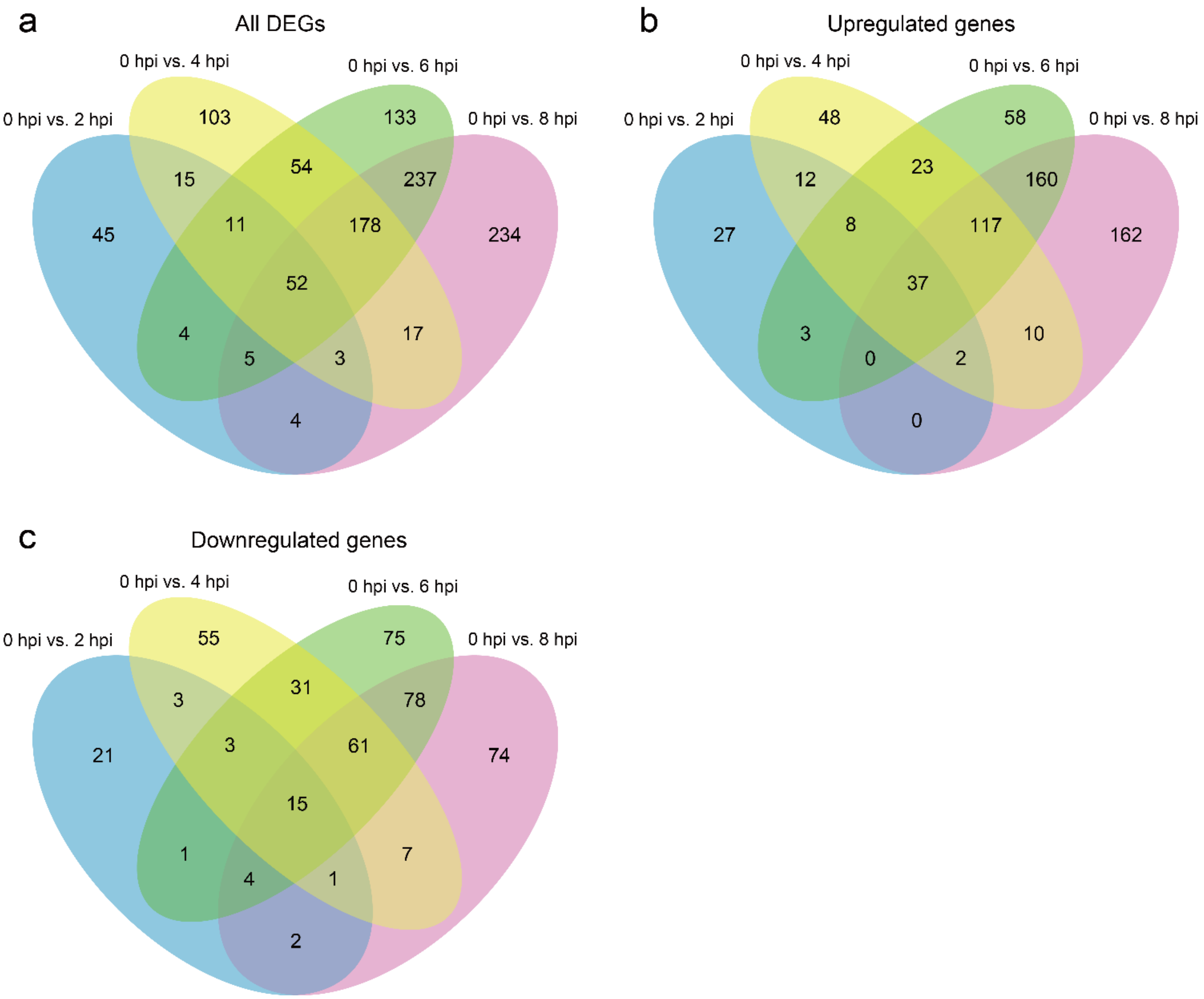

Supplementary figure 1. Distribution of differentially expressed (DE) genes in epithelial cells in response to adherent pneumococci at different infected time in comparison with mock infected time (0 hpi).

(**a**) Distribution of all DE genes in epithelial cells at different infected time in comparison with 0 hpi. (**b**) Distribution of upregulated genes at different infected time in comparison with 0 hpi. (**c**) Distribution of downregulated genes at different infected time in comparison with 0 hpi.

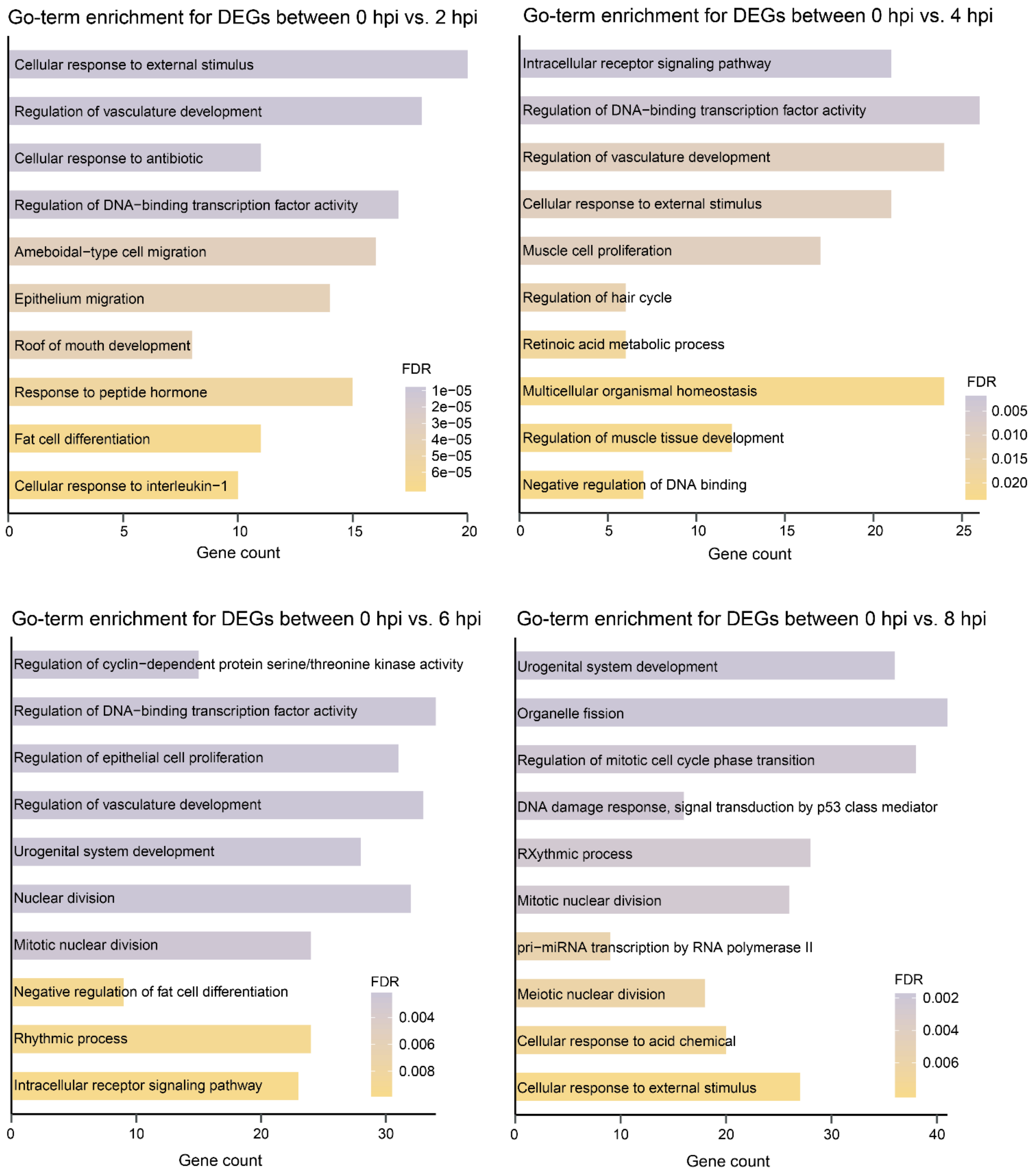

Supplementary figure 2. Gene Ontology analyses for differentially expressed genes at different infected time in comparison with 0 hpi.

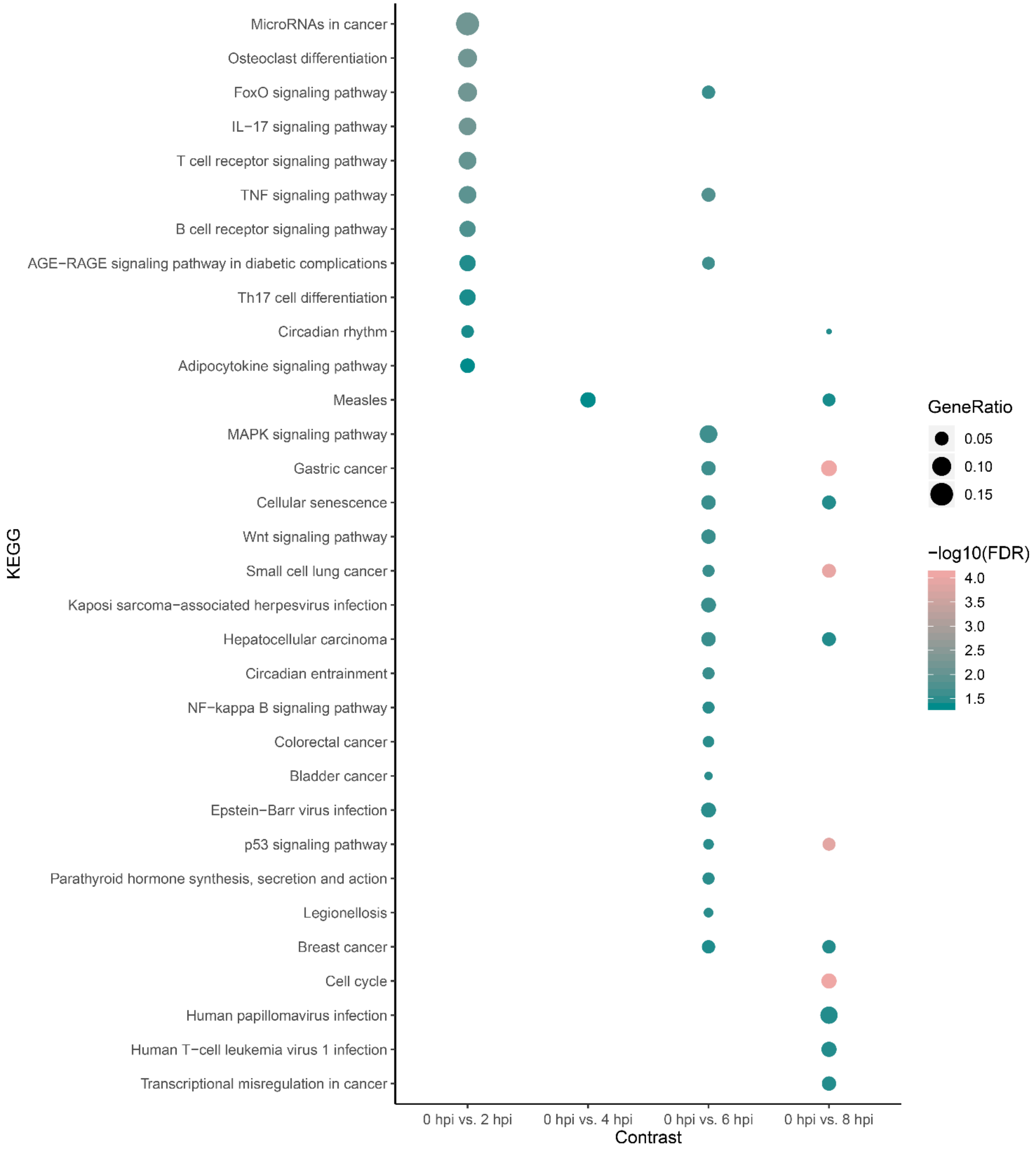

Supplementary figure 3. KEGG enrichment analyses for differentially expressed genes at different infected time in comparison with 0 hpi.

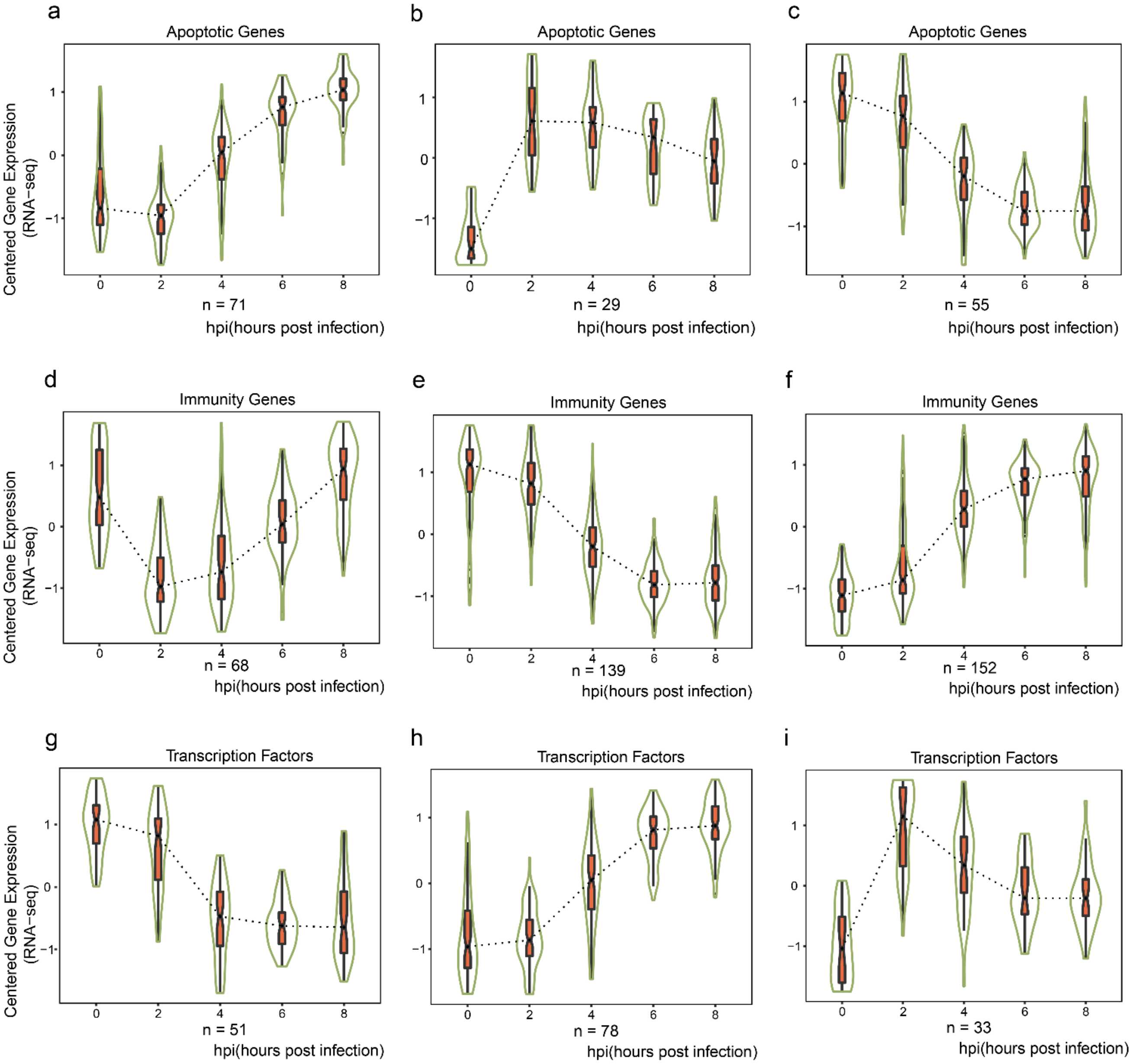

Supplementary figure 4. Unbiased automatic clustering of DE genes encoding apoptotic genes, immune factors, and transcription factors, utilizing the Mfuzz package in R.

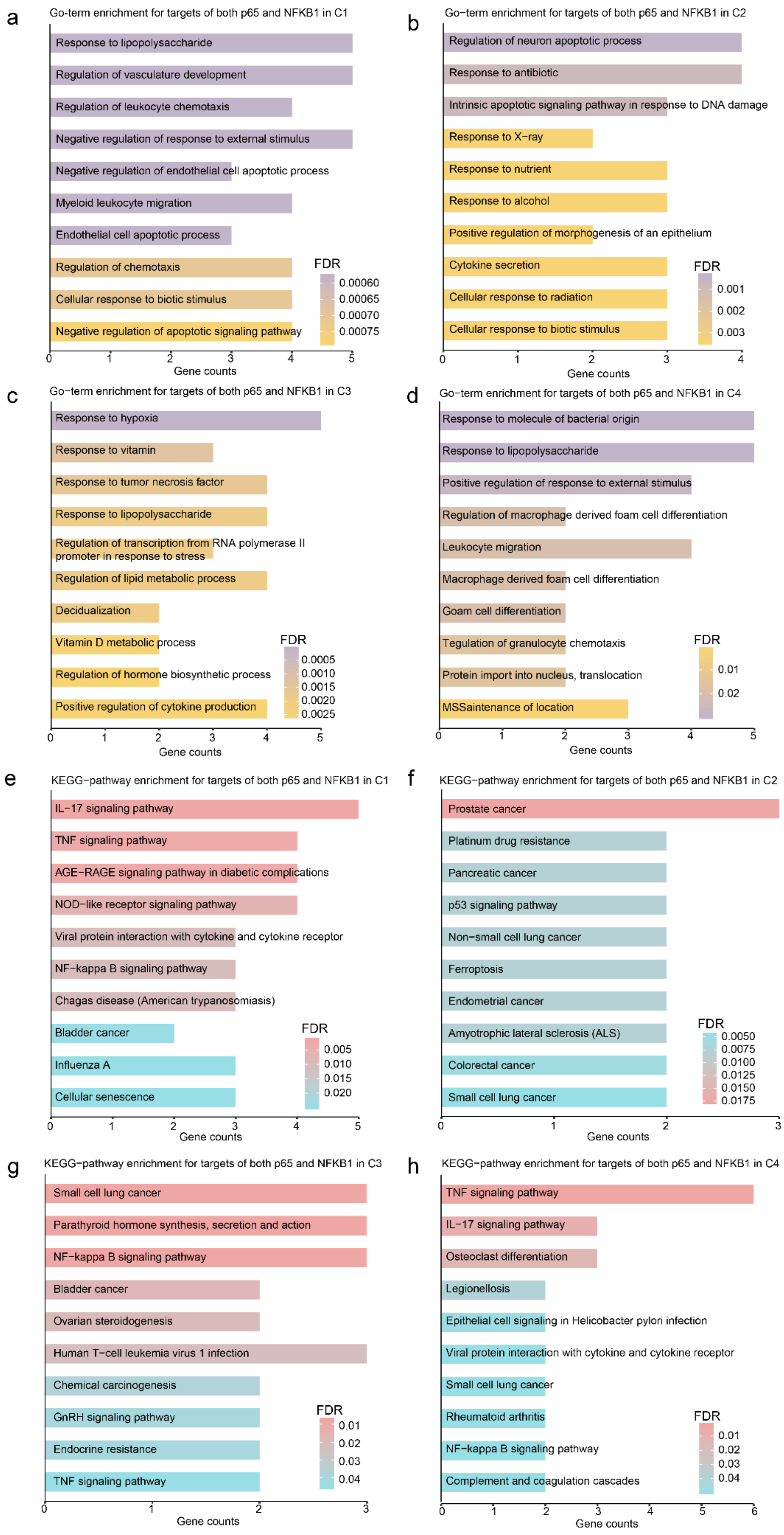

a
Go-term enrichment for targets of both p65 and NFKB1 in C1
Response to lipopolysaccharide
Regulation of vasculature development
Regulation of leukocyte chemotaxis
Negative regulation of response to external stimulus
Negative regulation of endothelial cell apoptotic process
Myeloid leukocyte migration
Endothelial cell apoptotic process
Regulation of chemotaxis
Cellular response to biotic stimulus
Negative regulation of apoptotic signaling pathway
FDR
0.00060
0.00065
0.00070
0.00075
0
1
2
3
4
5
Gene counts
b
Go−term enrichment for targets of both p65 and NFKB1 in C2
Regulation of neuron apoptotic process
Response to antibiotic
Intrinsic apoptotic signaling pathway in response to DNA damage
Response to X−ray
Response to nutrient
Response to alcohol
Positive regulation of morphogenesis of an epithelium
Cytokine secretion
Cellular response to radiation
Cellular response to biotic stimulus
FDR
0.001
0.002
0.003
0
1
2
3
4
Gene counts
c
Go−term enrichment for targets of both p65 and NFKB1 in C3
Response to hypoxia
Response to vitamin
Response to tumor necrosis factor
Response to lipopolysaccharide
Regulation of transcription from RNA polymerase II promoter in response to stress
Regulation of lipid metabolic process
Decidualization
Vitamin D metabolic process
Regulation of hormone biosynthetic process
Positive regulation of cytokine production
FDR
0.0005
0.0010
0.0015
0.0020
0.0025
0
1
2
3
4
5
Gene counts
d
Go−term enrichment for targets of both p65 and NFKB1 in C4
Response to molecule of bacterial origin
Response to lipopolysaccharide
Positive regulation of response to external stimulus
Regulation of macrophage derived foam cell differentiation
Leukocyte migration
Macrophage derived foam cell differentiation
Goam cell differentiation
Tegulation of granulocyte chemotaxis
Protein import into nucleus, translocation
MSSaintenance of location
FDR
0.01
0.02
0
1
2
3
4
5
Gene counts
e
KEGG−pathway enrichment for targets of both p65 and NFKB1 in C1
IL−17 signaling pathway
TNF signaling pathway
AGE−RAGE signaling pathway in diabetic complications
NOD−like receptor signaling pathway
Viral protein interaction with cytokine and cytokine receptor
NF−kappa B signaling pathway
Chagas disease (American trypanosomiasis)
Bladder cancer
Influenza A
Cellular senescence
FDR
0.005
0.010
0.015
0.020
0
1
2
3
4
5
Gene counts
f
KEGG−pathway enrichment for targets of both p65 and NFKB1 in C2
Prostate cancer
Platinum drug resistance
Pancreatic cancer
p53 signaling pathway
Non−small cell lung cancer
Ferroptosis
Endometrial cancer
Amyotrophic lateral sclerosis (ALS)
Colorectal cancer
Small cell lung cancer
FDR
0.0050
0.0075
0.0100
0.0125
0.0150
0.0175
0
1
2
3
Gene counts
g
KEGG−pathway enrichment for targets of both p65 and NFKB1 in C3
Small cell lung cancer
Parathyroid hormone synthesis, secretion and action
NF−kappa B signaling pathway
Bladder cancer
Ovarian steroidogenesis
Human T−cell leukemia virus 1 infection
Chemical carcinogenesis
GnRH signaling pathway
Endocrine resistance
TNF signaling pathway
FDR
0.01
0.02
0.03
0.04
0
1
2
3
Gene counts
h
KEGG−pathway enrichment for targets of both p65 and NFKB1 in C4
TNF signaling pathway
IL−17 signaling pathway
Osteoclast differentiation
Legionellosis
Epithelial cell signaling in Helicobacter pylori infection
Viral protein interaction with cytokine and cytokine receptor
Small cell lung cancer
Rheumatoid arthritis
NF−kappa B signaling pathway
Complement and coagulation cascades
FDR
0.01
0.02
0.03
0.04
0
1
2
3
4
5
6
Gene counts

Supplementary figure 5. Gene Ontology and KEGG enrichment analyses for differentially expressed (DE) genes regulated by p65 and NFKB1 in four clusters.

(**a-d**) The top 10 enriched GO terms of DE genes regulated by p65 and NFKB1 in four clusters. (**e-h**) The top 10 enriched KEGG terms of genes regulated by p65 and NFKB1 in four clusters.

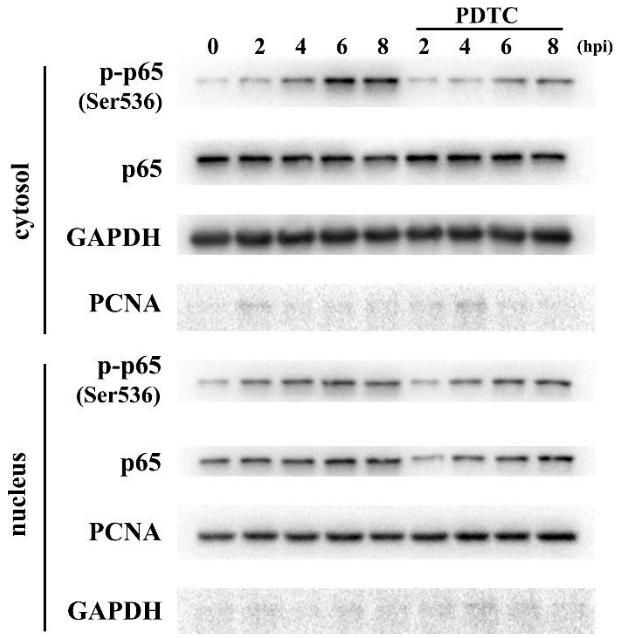

Supplementary figure 6. Protein levels of p65 and p-p65 were determined in nuclear and cytosolic fractions from A549 cells co-incubated with the *S. pneumoniae* with and without PDTC treatment for different infection times.

PCNA and GAPDH were shown as control for nucleus and cytosol, respectively.

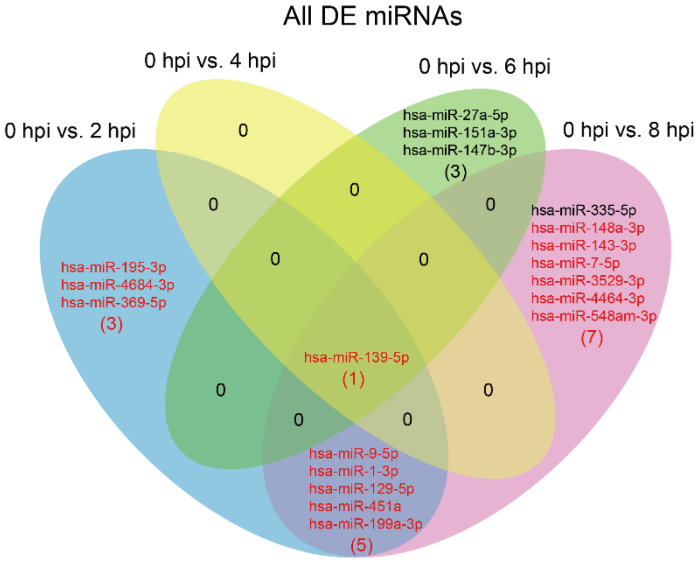

Supplementary figure 7. Distribution of differentially expressed miRNAs in epithelial cells in response to adherent pneumococci at different infected time in comparison with mock infected time (0 hpi).

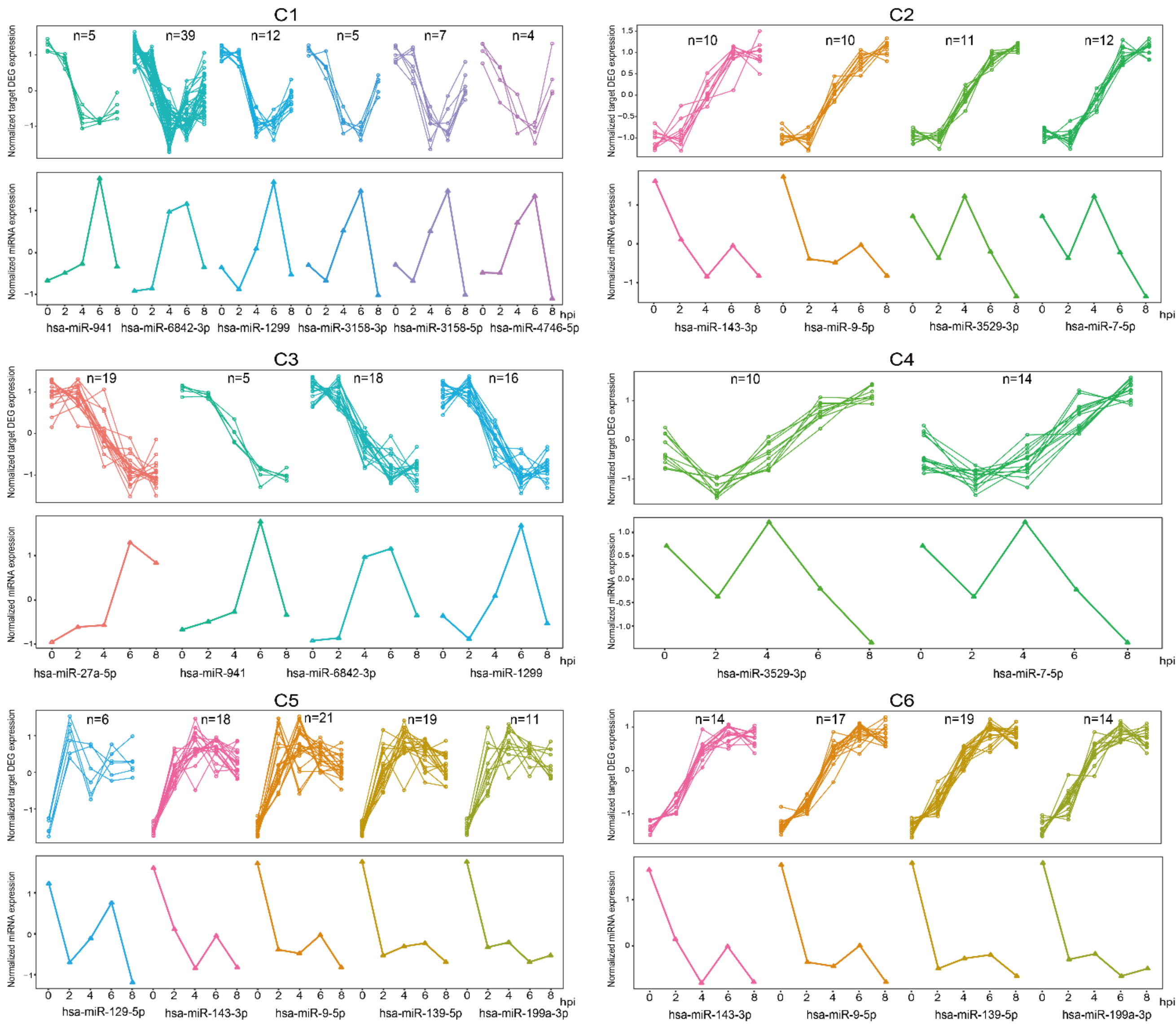

Supplementary figure 8. The expression trends of 14 DE-miRNAs and their target DE mRNAs along time series in the enriched clusters.

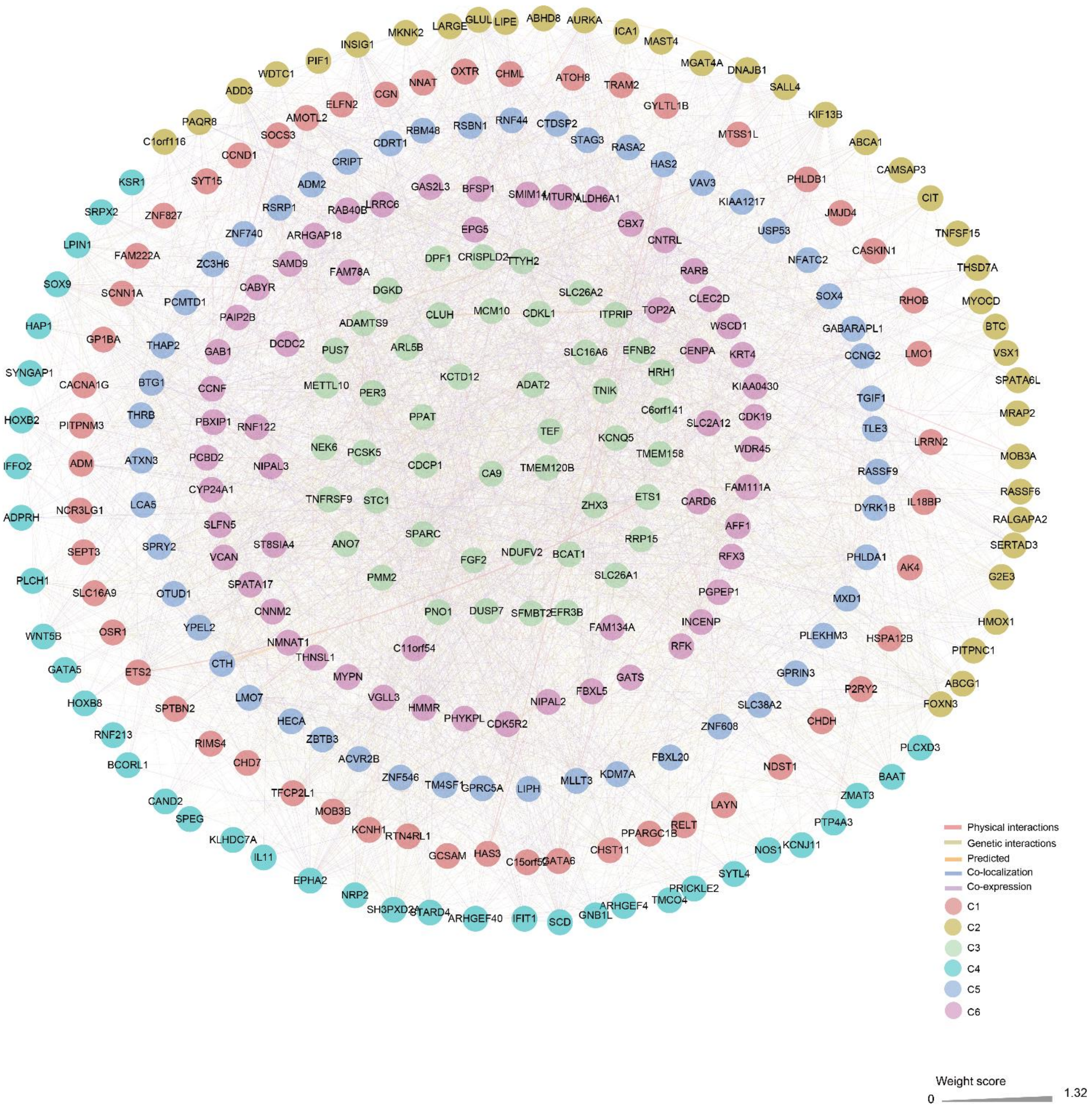

Supplementary figure 9. Huge complex biological network of all DE genes targeted by DE miRNAs using MCODE plug-in of Cytoscape.

This network includes 279 nodes (DE genes) and 3,700 edges (interactions). The colors of connections represent different interaction types. The filled color of each node represents the cluster to which the DE gene was assigned.

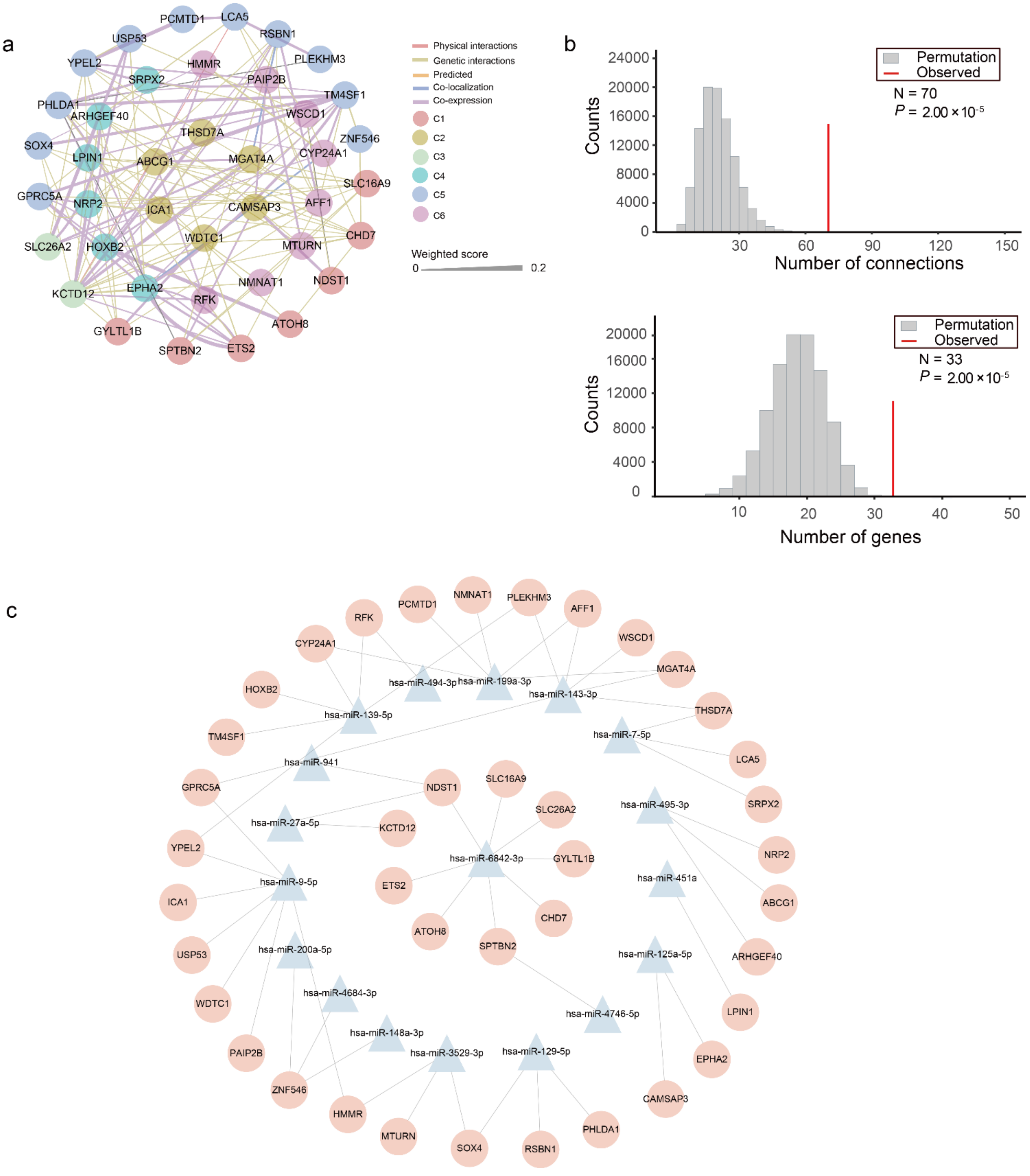

Supplementary figure 10. The regulatory relationship between miRNAs and target genes in module 2.

(**a**) Interaction network of module 2 (M2) identified by MCODE. The colors of connections represent different interaction types. The filled color of each node represents the cluster to which the DE gene was assigned. (**b**) A permutation test of M2 for genes (lower plot) and connections (upper plot) in

100,000 iterations was performed. The red line shows the number of genes or connections observed for the network of M2. (**c**) The network represents the regulatory relationship between miRNAs and target genes in M2. Triangular nodes represent miRNAs. The circular nodes represent the target genes regulated by miRNAs.

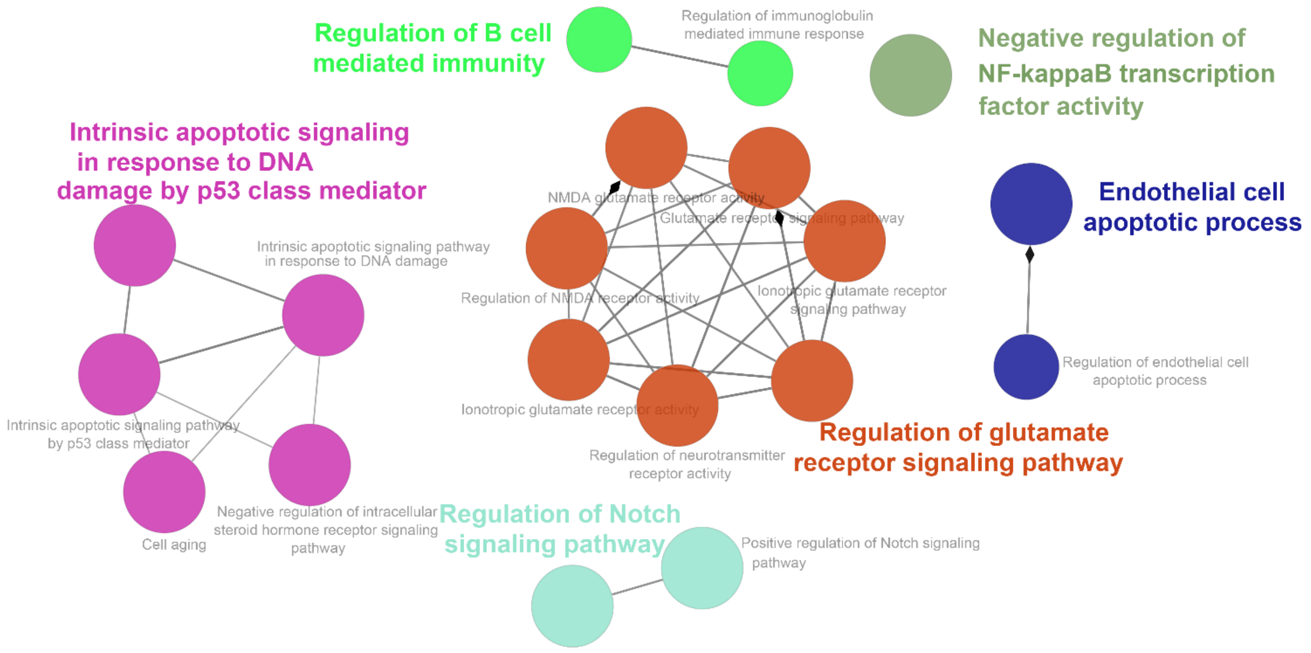

Supplementary figure 11. GO enrichment analysis of DE genes in module 1 using the "ClueGO" plug-in for Cytoscape.

Supplementary tables

Supplementary table 1. Evaluation of sequence data quality generated for cell transcriptome.

Time point	Sample ID	Raw reads	Clean reads	Clean bases	Q30 (%)	Mapped reads	Mapping rate
0 hpi	T0-1	34783274	32987414	9.9G	95.02	32158307	97.49%
	T0-2	22635683	22056986	6.62G	90.68	21016823	95.29%
	T0-3	28274346	27571236	8.27G	90.46	26237628	95.16%
2 hpi	T2-1	24327556	23722746	7.12G	90.51	22570021	95.14%
	T2-2	28097411	27128609	8.14G	94.24	26351758	97.14%
	T2-3	28261821	27305646	8.19G	94.11	26576579	97.33%
4 hpi	T4-1	26965357	26033778	7.81G	94.22	25333642	97.31%
	T4-2	26868579	25817711	7.75G	94.11	25071977	97.11%
	T4-3	23779010	22831607	6.85G	94.09	22163240	97.07%
6 hpi	T6-1	26273469	25218743	7.57G	94.26	24494559	97.12%
	T6-2	25325432	24314545	7.29G	94.18	23651487	97.27%
	T6-3	26033853	24994665	7.5G	94.19	24298361	97.21%
8 hpi	T8-1	25315568	24334373	7.3G	94.2	23645782	97.17%
	T8-2	24164996	22887991	6.87G	90.78	21761078	95.07%
	T8-3	30289524	29085598	8.73G	94.03	28321751	97.38%
Average		26759725	25752777	7.73G	93.27	24910200	96.68%

Definition of abbreviations: hpi, hours post infection.

Supplementary table 13. List of primers used for qRT-PCR analysis in this study.

Gene name	Primers (5'→ 3')	Length (bp)
CCNG2	Forward: TGGACAGGTTCTTGGCTCTT<br>Reverse: TCAACTATTCTAGCAGCCAGC	91
HMOX1	Forward: TAGAAGAGGCCAAGACTGCG<br>Reverse: CTTGGTGTCATGGGTCAGCA	80
REPS2	Forward: TGAACAGTGAGCTCCAGCAG<br>Reverse: ACCGGACGAAGTTGTTCCAA	85
ABCA1	Forward: AACCCCTGTTTCCGTTACCC<br>Reverse: AGCATCTGAGAACAGGCGAG	93
CCND1	Forward: AGCTGTGCATCTACACCGAC<br>Reverse: GAAATCGTGCGGGGTCATTG	113
NFKBIA	Forward: CCTACACCTTGCCTGTGAGC<br>Reverse: AGACACGTGTGGCCATTGTA	120
COL7A1	Forward: CAGCGACGTTCTACGGATCA<br>Reverse: TGGGAGTATCTGGTGCCTCA	106
TERT	Forward: GTTGGTGACACCTCACCTCA<br>Reverse: ACCACTGTCTTCCGCAAGTT	102
CCL2	Forward: GAAAGTCTCTGCCGCCCTT<br>Reverse: GGGGCATTGATTGCATCTGG	90
SIRT1	Forward: GAACATAGACACGCTGGAACAGGTT<br>Reverse: CTCCTCGTACAGCTTCACAGTCAAC	122
BCL3	Forward: ATTGCTGTGGTGCAGGGTAA<br>Reverse: ATGTGGTGATCACAGCCAGG	130
CSF1	Forward: AGCCACATGATTGGGAGTGG<br>Reverse: TTTGGCACGAGGTCTCCATC	76
CXCL1	Forward: TGCAGGGAATTCACCCCAAG<br>Reverse: ATGGGGGATGCAGGATTGAG	130
JUNB	Forward: CCACCTCCCGTTTACACCAA<br>Reverse: GAGGTAGCTGATGGTGGTCG	117
TRAF1	Forward: GGAAGCCGTCTTCGAACTCA<br>Reverse: AGGTGACCTCATGCTCTTGC	130

Supplementary table 2. List of DE genes in all comparisons (Excel file).

Supplementary table 3. Gene list for six clusters (Excel file).

Supplementary table 4. GO-term and KEGG-pathway enrichment for DE genes in all clusters (Excel file).

Supplementary table 5. GO-term and KEGG-pathway enrichment for DE genes at different infected time in comparison with 0 hpi (Excel file).

Supplementary table 6. The clusters for genes related to immunity, apoptosis, and human transcription factors (Excel file).

Supplementary table 7. Enrichment of DE genes regulated by key TFs in each cluster (Excel file).

Supplementary table 8. Summary of miRNA-seq (Excel file).

Supplementary table 9. Differentially expressed miRNAs in all comparisons (Excel file).

Supplementary table 10. Negative correlation between the expression level of miRNAs and mRNAs (Excel file).

Supplementary table 11. Enrichment of miRNA targets in each cluster (Excel file).

Supplementary table 12. GO-term enrichment of genes in Module 1 by "ClueGO" analysis (Excel file).
